# Flying with the birds? Recent large-area dispersal of four Australian *Limnadopsis* species (Crustacea: Branchiopoda: Spinicaudata)

**DOI:** 10.1002/ece3.265

**Published:** 2012-07

**Authors:** Martin Schwentner, Brian V Timms, Stefan Richter

**Affiliations:** 1 Universität Rostock, Allgemeine und Spezielle Zoologie, Institut für Biowissenschaften, Universitätsplatz 218055 Rostock, Germany; 2 Australian MuseumSydney, NSW 2000, Australia; 3 Australian Wetland and Rivers Centre, University of NSWSydney, NSW 2052, Australia

**Keywords:** Australia, Branchiopoda, dispersal, gene flow, temporary pools

## Abstract

Temporary water bodies are important freshwater habitats in the arid zone of Australia. They harbor a distinct fauna and provide important feeding and breeding grounds for water birds. This paper assesses, on the basis of haplotype networks, analyses of molecular variation and relaxed molecular clock divergence time estimates, the phylogeographic history, and population structure of four common temporary water species of the Australian endemic clam shrimp taxon *Limnadopsis* in eastern and central Australia (an area of >1,350,000 km^2^). Mitochondrial cytochrome c oxidase subunit I sequences of 413 individuals and a subset of 63 nuclear internal transcribed spacer 2 sequences were analyzed. Genetic differentiation was observed between populations inhabiting southeastern and central Australia and those inhabiting the northern Lake Eyre Basin and Western Australia. However, over large parts of the study area and across river drainage systems in southeastern and central Australia (the Murray–Darling Basin, Bulloo River, and southern Lake Eyre Basin), no evidence of population subdivision was observed in any of the four *Limnadopsis* species. This indicates recent gene flow across an area of ∼800,000 km^2^. This finding contrasts with patterns observed in other Australian arid zone taxa, particularly freshwater species, whose populations are often structured according to drainage systems. The lack of genetic differentiation within the area in question may be linked to the huge number of highly nomadic water birds that potentially disperse the resting eggs of *Limnadopsis* among temporary water bodies. Genetically undifferentiated populations on a large geographic scale contrast starkly with findings for many other large branchiopods in other parts of the world, where pronounced genetic structure is often observed even in populations inhabiting pools separated by a few kilometers. Due to its divergent genetic lineages (up to 5.6% uncorrected *p*-distance) and the relaxed molecular clock divergence time estimates obtained, *Limnadopsis parvispinus* is assumed to have inhabited the Murray–Darling Basin continuously since the mid-Pliocene (∼4 million years ago). This means that suitable temporary water bodies would have existed in this area throughout the wet–dry cycles of the Pleistocene.

## Introduction

In Australia's arid and semiarid zone, which covers about two-thirds of the continent (Martin 2006), permanent lakes are scarce and even river systems can be reduced to a series of permanent water holes for extended periods of time (Williams 1981; Faulks et al. 2010b). In these areas, temporary water bodies are important freshwater habitats. Various types of temporary water body can be distinguished, including turbid claypans, swamps with varying vegetation (e.g., black box or lignum swamps), large temporary lakes, and artificial farm dams (Williams 1981; Kingsford and Porter 1999). After rainfall, these pools usually fill from local runoff, but they may also be filled by flood or riverine water during severe flooding events and join up to form creeks or rivers for short periods of time (Timms 1999; Timms and Sanders 2002). The density of such pools across the landscape can be very high, as is the case in the catchment areas of the Paroo and Warrego Rivers in East Australia on the border between Queensland and New South Wales (part of the Murray–Darling Basin), where up to 26% of the land surface can be covered by claypans alone (Timms 1999). As a result, these areas are also important feeding and breeding grounds for water birds (Timms 1997; Kingsford and Porter 1999).

In addition to organisms, which also occur in other freshwater habitats, the fauna of temporary water bodies comprises a range of characteristic organisms (Timms 1999). A number of recent studies have investigated the phylogeography and genetic structure of aquatic organisms in Australia (reviewed in Hughes et al. 2009), but they all focused solely on taxa (fish, mollusks, and crustaceans) living in streams (Carini and Hughes 2004; Huey et al. 2006; Faulks et al. 2010a,b), permanent water holes (i.e., remnants of rivers during drought; Hughes et al. 2004; Carini and Hughes 2006), or permanent groundwater springs (Murphy et al. 2010). Strong genetic structuring with little to no gene flow was revealed in many species inhabiting groundwater springs or water holes (Hughes et al. 2004; Carini and Hughes 2006; Murphy et al. 2010), and riverine and some water hole species were usually structured genetically according to the main drainage systems (e.g., the Murray–Darling Basin, the Lake Eyre Basin, or the Bulloo), with no indication of ongoing gene flow across drainage system borders (Carini and Hughes 2004; Hughes et al. 2004; Huey et al. 2006; Faulks et al. 2010a,b). This is not surprising as these aquatic species can only disperse along interconnected waterways. However, all the studies in question neglected organisms that live exclusively in temporary water bodies, despite them being important components of the freshwater fauna of the arid zone of Australia (Kingsford and Porter 1999). Many of these organisms have resting or dormant stages, which survive the long drought periods in the sediment of dried-up pools. Among the most typical and common species in temporary pools are the “large branchiopod” crustaceans, namely tadpole shrimps (Notostraca), fairy shrimps (Anostraca), and clam shrimps (Spinicaudata and Laevicaudata), all of which produce “resting eggs” (actually cysts as these eggs are already fertilized) that need a desiccation period before hatching can be induced in a subsequent wet period (Brendonck 1996). Not only do resting eggs reestablish the active population within each pool, they are also the most important means of dispersal. Dispersal of resting eggs is always passive and may be mediated by wind, floods, or biotic vectors such as waterfowl (Bilton et al. 2001). In this latter case, the eggs either stick to the birds’ feathers or are carried internally after the ingestion of egg-bearing females. This mode of dispersal is not available to other aquatic taxa such as fish or mollusks and allows “large branchiopods” to disperse among unconnected water bodies. As aquatic species with resting eggs are generally among the first colonizers of new freshwater habitats, it has been assumed that the dispersal potential of resting eggs is high (Bilton et al. 2001). Most genetic studies, however, have revealed pronounced genetic differentiation even between populations inhabiting pools only a few kilometers apart (Vanoverbeke and De Meester 1997; Brendonck et al. 2000; Hulsmans et al. 2007; Thielsch et al. 2009; reviewed in De Meester et al. 2002). This dispersal-gene flow paradox has largely been ascribed to persistent founder effects (Boileau et al. 1992) coupled with the adaptive and numerical advantages of the local population, which together inhibit effective gene flow among populations (monopolization hypothesis; De Meester et al. 2002). Only a small proportion of the resting eggs hatch per wet period, resulting in a growing “resting egg bank” from which the subsequent generations are recruited (Brendonck and De Meester 2003). The resting egg bank acts as a buffer against invading genetic lineages. High dispersal rates, then, do not necessarily translate into high gene flow rates in these organisms (De Meester et al. 2002).

The evolution of species inhabiting the arid zone of Australia is closely coupled with the ongoing aridification, which started about 15 million years ago (mya) in the mid-Miocene (reviewed in Byrne et al. 2008 and Martin 2006). From then on to the end of the Pliocene (2.5 mya), arid zone taxa diversified, with most arid zone species dating to the Pliocene or even the mid-Miocene (Byrne et al. 2008). The transition from the Pliocene to the Pleistocene (2.5 mya) marked the initiation of glacial cycling, which corresponded in Australia with cold/dry glacial and warm/wet interglacial periods and an overall trend toward aridity. Within many arid zone species, the diversification of major genetic lineages occurred in the mid-Pleistocene (about 0.8 mya; reviewed in Byrne 2008), at which time shorter glacial cycles (∼40,000 years each) with lower amplitudes were replaced by longer cycles (∼100,000 years each) with higher amplitudes, coupled with an overall increase in aridity and an extensive expansion of arid environments (Byrne 2008).

The present study focuses on some of the most remarkable representatives of the Australian large branchiopods, those of the spinicaudatan taxon *Limnadopsis*. This taxon includes the world's largest clam shrimp *L. birchii*, which can reach up to 3 cm in length (Timms 2009). *Limnadopsis* species are restricted to temporary water bodies and never occur in permanent lakes or rivers. *Limnadopsis* is endemic in Australia, and its monophyly and Australian origin have been supported by recent molecular studies (Schwentner et al. 2009; Weeks et al. 2009). *Limnadopsis birchii* (Baird 1860) and *L. tatei*Spencer and Hall 1896 are found Australia-wide in arid and semiarid areas (Timms 2009). In an integrative taxonomic approach combining molecular and morpho-logical data, Schwentner et al. (2011) congruently delineated two species within *L. tatei*. One was identified as the true *L. tatei*Spencer and Hall 1896, which occurs in eastern, central, and western Australia (Schwentner et al. 2012), while the other, described as *L. paratatei*Schwentner et al. (2012), has so far only been reported from the catchment of the Paroo River in eastern Australia. *Limnadopsis parvispinus*Henry 1924 occurs from southern New South Wales to central and northern Queensland (Timms 2009; Schwentner et al. 2011). Schwentner et al. (2011) identified a clear genetic separation between the populations of the Murray–Darling Basin and the northeastern part of the Lake Eyre Basin, but suggested that they still represent a single species. (The lineages are referred to as *L. parvispinus*“Paroo” and “Buchanan,” respectively.)

In this paper, the phylogeography of four closely related *Limnadopsis* species is studied on the basis of two molecular markers (mitochondrial COI [cytochrome c oxidase subunit I] and nuclear ITS2 [internal transcribed spacer 2]) in order to assess the evolutionary and demographic history of species inhabiting Australian temporary water bodies. The main goal of this study is to identify the extent of population cohesion on various geographic scales (e.g., populations separated by a few kilometers versus those separated by more than 1000 km). Due to the patterns of genetic differentiation observed in populations of taxa with similar life-history traits in other parts of the world (e.g., other Branchiopoda with similar habitats, which are also dispersed via resting eggs), and those observed in species inhabiting isolated Australian freshwater systems (waterholes or groundwater springs), genetically differentiated populations are expected at geographic distances of much less than 100 km. Furthermore, a potential correlation existing between genetic structure and main drainage systems, as observed in many riverine species, is tested, because rare, severe floods may interconnect otherwise isolated temporary water bodies within river drainage systems, facilitating dispersal and gene flow within but not among drainage systems.

## Material and Methods

### Samples

**Figure 1:**
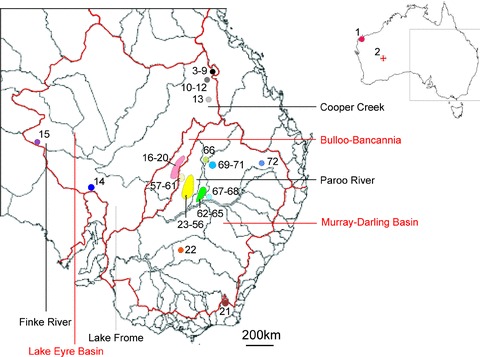
Geographic distribution of sample localities. The map shows main drainage systems (marked in red) and their subcatchments (marked in black). Each number represents a single sampled pool; further details of each locality are given in Table 1. Pools in close geographic proximity have been grouped together.

Adult specimens were collected between 1998 and 2011 (Fig. 1 and Table 1; Table S1). The specimens were fixed in absolute ethanol or RNAlater (Qiagen, Germany). In addition, samples of sediment from dry pools were collected. Small amounts of sediment (about 100 g) were incubated at 27°C in 2l glass aquaria filled with distilled water and subjected to a 16:8 light:dark cycle and constant aeration. Juveniles were reared on an algae suspension (Hobby-Liquizell®, Germany). If hatching success was low, single resting eggs were collected from the sediment sample for direct DNA extraction using a stereomicroscope (Olympus SZ51). All specimens were registered at the collection of the Australian Museum Sydney (see Table S1 for details).

**Table 1:** Details of sample localities. Drainage systems and locality numbers correspond to Figure 1

Drainage system	Locality	Locality description	Coordinates
Onslow Coast	1	CWP02, WA, 15.02.09	21°48′12.5″S, 115°06′01.4″E
Western Plateau	2	Lake Carey, WA, 08.03.2011	29°06′S, 122°17′E
Cooper Creek (LEB)	3	Y2, near Lake Buchanan, YS, QLD, 04.04.2009	21°30′55.2″S, 145°48′20.8″E
	4	Y7, near Lake Buchanan, YS, QLD, 24.02.2008	21°31′33.42″S, 145°48′6.96″E
	5	Y7 North, near Lake Buchanan, YS, QLD, 04.04.2009	21°34′34.1″S, 145°48′10.2″E
	6	Y12, near Lake Buchanan, YS, QLD, 04.04.2009	21°32′02.8″S, 145°48′15.6″E
	7a	Y17, near Lake Buchanan, YS, QLD, 04.04.2009	21°32′06.6″S, 145°47′50.7″E
	7b	Y17, near Lake Buchanan, YS, QLD, 26.02.2008	21°32′06.6″S, 145°47′50.7″E
	8	Y28, near Lake Buchanan, YS, QLD, 05.04.2009	21°31′06.1″S, 145°48′33.7″E
	9	Y31, near Lake Buchanan, YS, QLD, 05.04.2009	21°30′41.7″S, 145°48′09.5″E
	10	Lake Galilee, QLD, 15.02.2010	22°25′37.3″S, 145°42′13.4″E
	11	Pool next to Lake Dunn, QLD, 14.02.2010	22°36′16.4″S, 145°40′21.8″E
	12	Claypan near Lake Huffer, West of Lake Galilee, QLD, 28.02.2008	22°17′39.1″S, 145°21′22.1″E
	13	Swamp on Texas Station, QLD, 14.02.2010	23°02′37.5″S, 145°52′34.6″E
Lake Frome (LEB)	14	Clearwater Dug Out 113 km West Maree, SA, 12.3.2017	29°28′08.8″S, 137°01′35.5″E
Finke River (LEB)	15	Vegetated Stony Dug Out 34 km North Marla, SA, 10.3.2011	27°05′26.8″S, 133°28′16.2″E
Bulloo River	16	Yapunyah Pool, QLD, 28.2.2011	27°49′09.6″S, 144°09′26.5″E
	17	Swamp near Thargomindah Station, QLD, 26.2.2011	28°03′12.5″S, 143°47′11.5″E
	18	Flood Out of Dam, 84km South Thargomindah, QLD, 26.2.2011	28°39′46.7″S, 143°48′40.8″E
	19	Coolibah Swamp East of Road, QLD, 26.2.2011	28°50′47.6″S, 143°53′59.1″E
	20	Beefwood Grassy Swamp, QLD, 26.2.2011	28°50′34.5″S, 143°53′47.3″E
Snowy River	21	11 km North of Berridale, NSW, 14.03.2010	36°16′23.7″S, 148°48′13.6″E
Lachlan River (MDB)	22	Second East of Mossgiel, NSW, 23.01.2010; taken from sediment	33°17′43.2″S, 144°43′08.8″E
Paroo River (MDB)	23	Number 33 Black Box Swamp, RS, QLD, 09.06.2007	28°54′S, 144°58′E
	24	Poplar Box Pool, Western boundary fence, RS, QLD, 10.06.2007	28°56′S, 144°55.7′E
	25	Small Gilgai South of North Blue Lake, RS, QLD, 09.06.2007	28°51′S, 144°57.6′E
	26	Mid Blue lake, RS, QLD, 09.06.2007	28°53′S, 144°57′E
	27	Coolibah Swamp, RS, QLD, 01.04.2009	28°54′03.2″S, 144°59′22.6″E
	28	Beverley's Pool, BS, NSW, 19.02.2010	29°32′12.0″S, 144°51′16.1″E
	29	Dead Ram Pan, BS, NSW, 21.2.2011	29°31′45.4″S, 144°52′05.4″E
	30	Western Fence, BS, NSW,.03.2006; taken from sediment	29°24′58.4″S, 144°46′52.8″E
	31	Gidgee Lake, BS, NSW, 19.02.2010	29°33′10.4″S, 144°50′12.7″E
	32	Homestead Swamp 1, BS, NSW, 19.02.2010	29°31′31.5″S, 144°53′27.7″E
	33	Lower Crescent Pool, BS, NSW, 19.01.2010	29°32′34.5″S, 144°51′31.6″E
	34	Upper Crescent Pool, BS, NSW, 19.01.2010	29°32′33.6″S, 144°52′16.5″E
	35	Roskos Paleolake, BS, NSW, 19.02.2010	29°27′42.9″S, 144°48′12.5″E
	36	Titanic Black Box Swamp, BS, NSW, 07.06.2007	29°26′S, 144°47′E
	37a	Marsilea, BS, NSW, 19.02.2010	29°32′13.2″S, 144.52′26.3″E
	37b	Marsilea, BS, NSW, 21.02.2011	29°32′13.2″S, 144.52′26.3″E
	38	Vosper Pool, BS, NSW, 19.01.2010	29°32′03.9″S, 144°50′37.7″E
	39a	Carters Swamp, MS, NSW, 28.07.2009; raised from sediment	29°26′05 ″S, 144°58′52 ″E
	39b	Carters Swamp, MS, NSW, 1999; taken from sediment	29°26′05 ″S, 144°58′52 ″E
	39c	Carters Swamp, MS, NSW, 19.01.2010	29°26′05 ″S, 144°58′52 ″E
	40	Carols Bore, MS, NSW, 19.01.2010	29°29′08.9″S, 144°59′13.4″E
	41	small lake on East Boundary, north side of road, MS, NSW, 20.02.2010	29°31′38.5″S, 145°00′43.7″E
	42	small lake on East Boundary, south side of road, MS, NSW, 20.02.2010	29°31′54.1″S, 145°01′02.4″E
	43	Lismore Bore, MS, NSW, 19.01.2010	29°31′50.7″S, 144°59′28.1″E
	44	Lower Lake Eliza, MS, NSW, 20.02.2010	29°25′28.9″S, 145°03′41.8″E
	45	Yungerina Black Box Swamp, MS, NSW, 20.01.2010	29°26′09.1″S, 145°04′40.3″E
	46	Pool on Yungerina, MS, NSW, 20.01.2010	29°28′14.8″S, 145°06′27.2″E
	47	Muella Vegetated Pool 1, MS, NSW, 31.03.2009	29°31′10.3″S, 144°56′21.8″E
	48	Muella Vegetated Pool 2, MS, NSW, 31.03.2009	29°31′00.3″S, 144°56′22.7″E
	49	Muella Vegetated Pool 3, MS, NSW, 31.03.2009	29°30′12.0″S, 144°55′37.4″E
	50	Muella Vegetated Pool 4, MS, NSW, 31.03.2009	29°30′00.7″S, 144°54′59.6″E
	51	Quandong Swamp, TS, NSW, 23.2.2011	29°27′45.4″S, 144°51′06.6″E
	52	Tredega Mail Box Black Box Swamp, TS, NSW, 21.2.2011	29°29′26.1″S, 144°52′06.4″E
	53	Yantabulla Black Box Swamp, NSW, 31.03.2009	29°20′18.0″S, 145°00′12.1″E
	54	Grassy Pool North of Yantabulla, NSW, 20.01.2010	29°19′04.8″S, 145°00′31.5″E
	55	Big Black Box Swamp, NSW, 21.01.2010	29°10′19.4″S, 145°22′41.3″E
	56	Black Box Swamp near Cumeroo, NSW, 20.01.2010	29°15′41.2″S, 145°09′29.0″E
	57	Well Vegetated Claypan, CNP, QLD, 24.2.2011	28°47′19.4″S, 144°17′43.3″E
	58	Claypan North of Windmill, CNP, QLD, 24.2.2011	28°48′28.8″S, 144°18′09.1″E
	59	Bokeen Cane GrasSwamp, CNP, QLD, 24.2.2011	28°49′55.3″S, 144°20′59.3″E
	60	Grassy Pool at Northern Fence, CNP, QLD, 25.2.2011	28°52′20.2″S, 144°20′47.9″E
	61	Big Darko Claypan, CNP, QLD, 25.2.2011	28°52′19.1″S, 144°17′34.5″E
Warrego River (MDB)	62	Gerara Composite Swamp, NSW, 21.01.2010	29°11′47.0″S, 146°17′03.0″E
	63	Pool South of Gerara, NSW, 21.01.2010	29°13′51.4″S, 146°18′22.6″E
	64	Claypan-like West of Engonia, NSW, 21.01.2010	29°18′32.8″S, 145°44′06.9″E
	65a	East of Lake Lauradale, NSW, 29.03.2009	29°51′22″S, 145°38′49 ″E
	65b	East of Lake Lauradale, NSW, 18.01.2010	29°51′22″S, 145°38′49 ″E
	66	North Wyandra, QLD, 17.02.2010	27°11′03.2″S, 145°59′41.2″E
Condamine-Culgos River (MDB)	67	Roadside Claypan, NSW, 21.01.2010	29°31′42.5″S, 146°12′20.5″E
	68	Turbid Pool, NSW, 21.01.2010	29°32′29.3″S, 146°24′50.1″E
	69	Swamp 94 km East Wyandra, QLD, 17.02.2010	27°20′50.7″S, 146°47′35.8″E
	70	Cyclestheria Grassy Swamp, QLD, 18.02.2010	27°40′48.8″S, 146°38′02.7″E
	71	Grassy Turbid Swamp, QLD, 18.02.2010	27°41′52.4″S, 146°45′44.7″E
	72	Big Pool on Meandarra Road, QLD, 12.02.2010	27°22′43.9″S, 150°01′18.1″E

BS, Bloodwood Station; CNP, Currawinya National Park; LEB, Lake Eyre Basin; MDB, Murray–Darling Basin; MS, Muella Station; NSW, New South Wales; RS, Rockwell Station; QLD, Queensland; SA, South Australia; TS, Tredega Station; WA, Western Australia; YS, Yarromere Station.

### DNA extraction, PCR amplification, and sequencing

DNA extraction followed the HotShot protocol (Montero-Pau et al. 2008). Small fractions of the musculature connecting the carapace halves were incubated in 40 µL lysis buffer for 30 min at 95°C and then mixed with 20 µL HCl buffer. For the PCR amplification of the COI fragment, several primers were used in varying combinations, always featuring one LCO and one HCO primer: LCO1490 5′-GGT CAA CAA ATC ATA AAG ATA TTG G-3′ (Folmer et al. 1994), LCO2 5′-TCN ACH AAY CAT AAA GAY ATT GGA AC-3′ (new primer designed by L. Krebes and R. Bastrop), LCO3 5′-TCN ACH AAY CAT AAA GAY ATT GGT AC-3′ (Krebes et al. 2010), HCOoutout 5′-GTA AAT ATA TGN TGN GCT C-3′ (Folmer et al. 1994), HCO-MZ1-rev 5′-CTT TVA TDC CNG TVG GSA CWG CRA TAA TYA T-3′ (Krebes et al. 2010), and HCO709 5′-AAT NAG AAT NTA NAC TTC NGG GTG-3′ (Blank et al. 2008). The ITS2 sequence was amplified in full using the primers ITS3 5′-GCA TCG ATG AAG AAC GCA GC-3′ (White et al. 1990) and ITS28 5′-CGC CGT TAC TAG GGG AAT CCT TGT AAG-3′ (Wagstaff and Garnock–Jones 1998). PCR reactions contained 3 µL of each primer, 0.15 µL MolTaq polymerase (Molzym), 3 µL 10× buffer, 3 µL dNTPs (2 mM), 0.8 µL MgCL_2_ (50 mM) topped up with H_2_O to a total of 30 µL. The PCR program for COI consisted of an initial denaturation step of 1 min at 94°C, followed by 38 amplification cycles (94°C for 1 min, 46°C for 30 sec, 70°C for 1 min), and a final elongation step at 70°C for 5 min. For the amplification of ITS2, the annealing temperature was set to 50°C. PCR products were purified using magnetic beads (Agencourt AMPure, Beckman Coulter, Germany) or by cutting out visible bands using the QIAquick Gel Extraction Kit (Qiagen, Germany) following the manufacturers’ instructions. PCR products were sequenced with the DCTS Quick Start Kit (Beckman Coulter) on an automated sequencer (CEQTM 800 from Beckman Coulter) or by the Qiagen Sequencing Service (Qiagen). All in all, 413 specimens were sequenced for COI and a subset of 63 specimens for ITS2. Sequence electropherograms were analyzed using Sequencher 4.1.4 (Gene Codes), and corrected sequences were aligned using the ClustalW algorithm (Thompson et al. 1994) as implemented in Bioedit (Hall 1999). All sequences were submitted to GenBank (see Table S1 for details).

### Population indices and geographic structure of populations

In the following, certain terms are used to describe the hierarchical structure of geographic regions and populations. The smallest unit-population refers to the individuals living in the same pool. The term (sub)catchment refers to the catchment area of single rivers (e.g., the Cooper or the Paroo), whereas (main) drainage system denotes larger areas usually consisting of several interconnected (sub)catchments (e.g., the Murray–Darling and Lake Eyre Basins or the Bulloo River). In Figure 1 the drainage systems are marked in red and the catchments are marked in gray. As Schwentner et al. (2011) referred to the two main *L. parvispinus* lineages as “Paroo” and “Buchanan,” we will use the same terminology for *L. parvispinus* and extend it to *L. birchii* as well. “Paroo” and “Buchanan” thus refer to populations featuring certain geographically restricted genetic lineages. “Paroo” relates to populations in southern Queensland and New South Wales (covering the whole Murray–Darling Basin and the Snowy River catchment; in the case of *L. birchii* extending west to the Bulloo and the southern Lake Eyre Basin), whereas “Buchanan” relates to populations in central and northern Queensland (northern part of the Cooper Creek catchment, which belongs to the Lake Eyre Basin).

Owing to the more extensive COI dataset, most of the following analyses were performed solely with this gene fragment, meaning that most results and conclusions will be based on COI. To avoid repetition, COI will not be referred to in all instances.

The number of variable and parsimony-informative sites was computed using Mega5 (Tamura et al. 2011) for each gene and species separately. The standard population genetic indices of haplotype and nucleotide diversity with their respective standard deviations were calculated for COI using the program Arlequin 3.5 (Excoffier and Lischer 2010).

To assess the geographic distribution of genetic lineages, COI and ITS2 haplotype networks were calculated using the Median Joining algorithm in Network 4.6 (Fluxus Techno-logies Ltd.). One single network was produced for ITS2 while for COI a separate network was calculated for each species. A fundamental assumption underlying these network analyses (and most of the following phylogeographic analyses) is that each haplotype evolved only once so occurrence of the same haplotype in different populations is due to historical dispersal events. Schwentner et al. (2011) identified two highly divergent lineages within *L. parvispinus* with uncorrected *p*-distances in COI of up to 9.9%. As the genetic distance between these two lineages might affect the topology within them, Median Joining networks were calculated for each lineage independently as well as for the whole *L. parvispinus* dataset.

To determine whether or not populations are structured according to drainage systems, several analyses of molecular variance (AMOVAs; Excoffier et al. 1992) were performed using Arlequin 3.5, with different groupings for each species (COI only). AMOVA is a method used to calculate genetic subdivision among and within different hierarchical levels. Only populations (pools) from which at least four individuals were available were included (the only exception being the *L. birchii* population from Onslow, Western Australia, from which only three individuals were available), as populations of fewer individuals could obscure the results. For each species, one AMOVA was run, with populations grouped according to subcatchments within drainage systems. Additional AMOVAs were performed for *L. birchii* and *L. parvispinus* in which the populations were grouped according to the main genetic lineages. For *L. birchii*, three groups were predefined: (1) all populations of the Murray–Darling Basin, the Bulloo River, and the Finke River (northwestern New South Wales, southeastern Queensland, and South Australia), (2) Western Australia, and (3) the Cooper Creek (central Queensland). For *L. parvispinus*, two additional AMOVAs were run. In the first, two groups were predefined, which correspond to the two main genetic lineages “Paroo” and “Buchanan” described by Schwentner at al. (2011). In the second run, only populations of the “Paroo” lineage were included. The grouping here corresponded to the subcatchments of this drainage system. The statistical significance of each AMOVA was assessed by 1000 permutations.

Because the predefined population groupings used in the AMOVA analyses might not be representative of actual genetic structuring within the species, we also conducted a spatial analysis of molecular variance (SAMOVA; Dupanloup et al. 2002), which does not require predefined groups of populations but itself comes up with geographically homogeneous groups of populations that are maximally genetically differentiated from each other (Dupanloup et al. 2002). The ideal grouping was determined by running SAMOVA several times for each species with the number of inferred groups ranging from two to 10. The grouping, which resulted in the highest *F*_CT_ value (variation among groups), is, in theory, that which represents the maximum genetic differentiation among groups of populations. Only those populations included in the AMOVA runs were used in the SAMOVAs. For *L. parvispinus*, additional SAMOVA runs were carried out for each of the two main genetic lineages.

To assess the degree of population differentiation, pairwise Φ_ST_ for COI was calculated using Arlequin 3.5. Here again, only populations for which at least four individuals were available were included. In contrast to traditional *F*_ST_, which only considers haplotype frequencies, Φ_ST_ also takes the differences between haplotypes into account. Mantel tests for each species were performed in Arlequin 3.5 to determine whether genetic differentiation is correlated to geographic distance (isolation-by-distance). Pairwise Φ_ST_ values were compared to the geographic distance in kilometer. Statistical significance was tested by 1000 permutations.

### Demographic history

The demographic history (e.g., population expansion or bottleneck events) of the four *Limnadopsis* species was determined by means of neutrality tests and COI data mismatch distributions carried out in Arlequin 3.5. To test whether populations evolve under neutrality, Fu's *F*_s_ (Fu 1997) and Tajima's *D* (Tajima 1989) were calculated. In both tests, positive values are indicative of mutation-drift-equilibrium, which is typical of stable populations. Negative values result from an excess of rare haplotypes, a condition typical of populations that have undergone recent expansions, often preceded by a bottleneck. During expansion events, the mutation rate exceeds the rate of extinction (drift), leading to a proportionally high number of haplotypes. Significantly negative values (at the 0.05 level) reveal in both tests historic demographic expansion events. Significance was tested by 1000 permutations.

Mismatch distributions, which represent the distribution of pairwise nucleotide differences between all the individuals in a given dataset and are used to test for sudden expansion events and infer their relative timing, were calculated using the sudden expansion model. The validity of the sudden expansion assumption was determined using the sum of squared deviations (SSD) and Harpending's raggedness index (Hri; Harpending 1994), both of which are higher in stable, nonexpanding populations (e.g., those with a multimodal mismatch distribution). Significant (*P*≤ 0.05) SSD and Hri values mark deviations from the sudden expansion model. Conversely, sudden expansion cannot be rejected if SSD and Hri values are nonsignificant (*P* > 0.05).

### Genetic distances and divergence times

Pairwise genetic distances between sequences within each species and within and between the main genetic lineages were calculated for both genes as uncorrected *p*-distances (i.e., the actual difference in percent) using Mega5. To estimate the divergence times of different COI lineages, Kimura-2-Parameter (K2P) corrected distances were determined for the COI dataset. Divergence time estimates are, in the absence of reliable estimates of COI divergence rates specifically for branchiopods, based on the suggested decapod crustacean pairwise divergence rates of 1.4% (Knowlton and Weigt 1998) and 2.3% per million years (Schubart et al. 1998). Cristescu et al. (2003) took the same approach. It has been assumed that resting egg banks tend to stabilize populations and thus reduce evolutionary rates (Hairston and De Stasio 1988), therefore it is presumed that the divergence rate for branchiopods is closer to the lower bound of 1.4% per million years. All time estimates should be regarded with great caution as long as no branchiopod-specific divergence rates are available. Divergence times were not estimated for the ITS2 dataset as rate calibration is not available for crustaceans.

Phylogenetic analyses coupled with divergence time estimates for COI were performed in BEAST v1.6.1 (Drummond and Rambaut 2007) using the *BEAST (Heled and Drummond 2010) option, which permits the reconstruction of divergence times using numerous individuals of different species. An uncorrelated relaxed clock (Drummond et al. 2006) with log-normal rate distribution among branches was applied using the Yule speciation model with default settings. The substitution rate was set to 1 to obtain age estimates in units of substitutions per site under the HKY substitution model. The best-fitting substitution model had been determined in Mega5. (The T92 model was the best-fitting model, but the HKY was chosen as it is the most similar model available in BEAST). The substitutions per site were translated into age estimates in units of million years on the basis of the divergence rate estimates of 1.4% and 2.3% per million years (prioritizing the 1.4% estimate; see above.) Two independent runs each of 30*10^6^ generations were performed, sampling one tree every 3000 generations. For all parameters, convergence in the two runs and an effective sample size (ESS) of over 200 was determined using Tracer 1.5 (Rambaut and Drummond 2004). The resulting tree files were combined in LogCombiner v1.6.1 (Drummond and Rambaut 2007) and analyzed in TreeAnnotator v1.6.1 (Drummond and Rambaut 2007), discarding the first 10% of the sampled trees as burn-in. Visualization of trees and determination of the age of respective branches was performed in Figtree v1.2 (Rambaut 2008).

## Results

### Population indices and geographic structure of populations

The alignment of the COI fragments included a total of 413 sequences (Table 2) and was 519 bp in length without indels. The derived amino acid sequence contained no stop codons, thus providing no indication of nuclear paralogs. The number of variable and parsimony-informative sites was 133 and 84, respectively, for *L. parvispinu*s, 50 and 35 for *L. birchii*, 12 and two for *L. tatei*, and 12 and 11 for *L. paratatei*. The ITS2 alignment was 689 bp in length with 98 variable and 80 parsimony-informative sites. In addition, 64 sites featured indels, accounting for more than a third of the total variation observed. Most indels occurred between different species. The longest indel was 8 bp in length, while most were 1 or 2 bp long. Several sequences contained ambiguous sites, most likely indicating the presence of different alleles within individuals and thus suggesting that the genetic diversity within the ITS2 dataset may have been underestimated. Additional haplotypes not displayed in the haplotype network (Fig. 3) may have been present.

**Table 2:** Genetic indices, parameters of demographic history and Mantel tests for all four *Limnadopsis* species and the main lineages within species inferred from cytochrome c oxidase subunit I

							Mismatch distribution (demographic expansion)			Mantel test^+^	
								
	# ind.	# hapl.	π± SD	h ± SD	Fu's *F*_s_ (*P*-value)	Tajima's *D* (*P*-value)	τ (relative measure of time in generations)	Sum of squared deviations (*P*-value)	Harpending's raggedness index (*P*-value)	rY1 correlation coefficient (*P*-value)	Determination of Y1 (Φ_ST_) by X1 (distance in km) in percentage
*L. parvispinus* (all)	167	72	0.0442 ± 0.0217	0.956 ± 0.010	−11.755 (0.053)	1.215 (0.91)	47.480	0.01313 (0.30)	0.00811 (0.15)	0.8139 (0.00)	66.2
*L. parvispinus*“Paroo”	127	55	0.0200 ± 0.0102	0.929 ± 0.017	−19.850 (0.00)	−0.552 (0.34)	17.438	0.01291 (0.43)	0.01172 (0.54)	0.5796 (0.06)	33.6
*L. parvispinus*“Buchanan”	40	17	0.0121 ± 0.0065	0.945 ± 0.020	−3.830 (0.10)	−0.274 (0.46)	7.902	0.00485 (0.78)	0.01308 (0.71)	0.8657 (0.01)	74.9
*L. birchii* (all)	154	37	0.0087 ± 0.0048	0.923 ± 0.010	−15.889 (0.00)	−1.503 (0.03)	0.906	0.18009 (0.00)	0.01639 (1.00)	0.6177 (0.00)^++^	38.2^++^
*L. birchii*“Paroo”	129	32	0.0045 ± 0.0027	0.906 ± 0.014	−24.483 (0.00)	−1.878 (0.01)	2.479	0.00056 (0.65)	0.03022 (0.57)	0.1490 (0.10)	2.2
*L. birchii*“Buchanan”	21	4	0.0010 ± 0.0010	0.471 ± 0.116	−1.456 (0.04)	−1.007 (0.18)	0.645	0.01254 (0.28)	0.16061 (0.25)	0.8484 (0.16)	72
*L. tatei*	48	8	0.0019 ± 0.0015	0.511 ± 0.081	−3.028 (0.03)	−1.866 (0.01)	1.020	0.00136 (0.90)	0.07847 (0.84)	−0.0166 (0.53)	0.03
*L. paratatei*	43	6	0.0067 ± 0.0038	0.745 ± 0.040	3.136 (0.89)	0.727 (0.80)	10.0	0.07377 (0.10)	0.16252 (0.02)	−0.0365 (0.21)	0.1

“Paroo” denotes populations from the Murray–Darling Basin, the Bulloo and southern parts of the Lake Eyre Basin; “Buchanan” denotes populations from northern parts of the Lake Eyre Basin (Cooper Creek). # ind., number of individuals; # hapl., number of haplotypes; π, nucleotide diversity; h, haplotype diversity;^+^only populations with at least four sequences were included;^++^ the Western Australian population was included though only three sequences were available.

*Limnadopsis parvispinus* and *L. birchii* have a large number of different COI haplotypes, which is reflected in high haplotype diversities of >0.9 (Table 2). In *L. parvispinus*, the high haplotype diversity correlates with a high nucleotide diversity of 0.0442, over five times greater than the nucleotide diversity of the other three species. The high nucleotide diversity of *L. parvispinus* reflects the genetic differentiation between the two main lineages “Paroo” and “Buchanan.” While nucleotide diversity within the lineages themselves is lower, at 0.020 it is still higher in the “Paroo” lineage than in any of the other species. The overall nucleotide diversity of 0.0087 in *L. birchii* is lower than in *L. parvispinus. Limnadopsis tatei* and *L. paratatei* have relatively few closely related haplotypes (Table 2). As specimens from a wide range of localities in central and eastern Australia were sampled, the low nucleotide diversities most likely reflect the species’ true diversity within this area and are not an artifact caused by smaller sample size. Genetic diversity in Western Australia cannot be assessed as only a single specimen of *L. tatei* was studied from this area.

**Figure 2:**
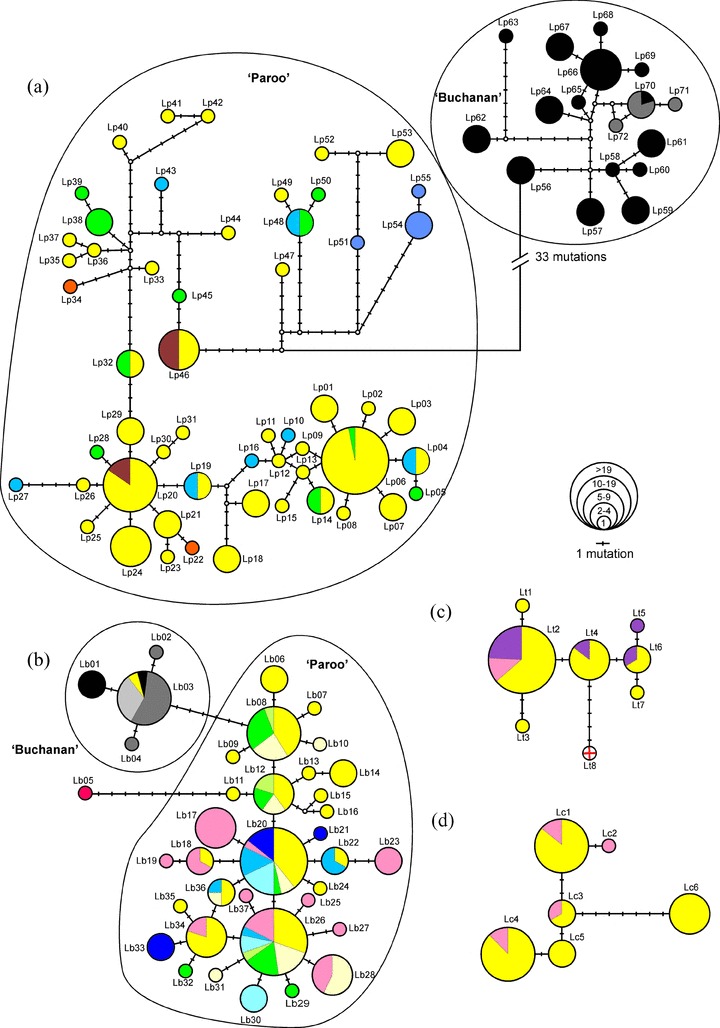
Median joining cytochrome c oxidase subunit I haplotype networks for all four *Limnadopsis* species. (a) *Limnadopsis parvispinus*, (b) *L. birchii*, (c) *L. tatei*, and (d) *L. paratatei*. Each circle represents a single haplotype with the diameter of the circle corresponding to the number of individuals sharing the haplotype (see also Tables S2–S5). The colors represent the respective sample localities (see Fig. 1 for details).

**Figure 3:**
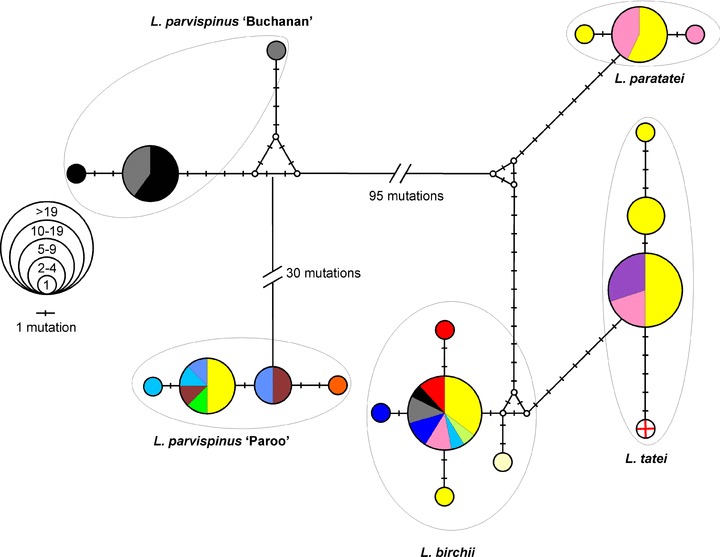
Median joining internal transcribed spacer 2 haplotype network for all four *Limnadopsis* species. Indels (gaps in the alignment) are accounted for as mutational steps, so the number of mutational steps may differ from those deduced from the genetic distances shown in Table 5. Each circle represents a single haplotype with the diameter of the circle corresponding to the number of individuals sharing the haplotype. The colors represent the respective sample localities (see Fig. 1 for details).

**Figure 4:**
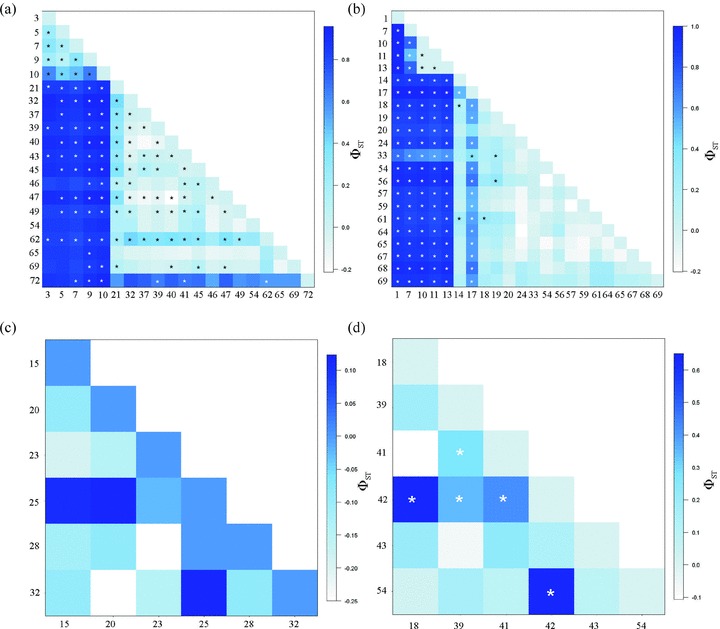
Pairwise Φ_ST_ values for cytochrome c oxidase subunit I between populations of (a) *Limnadopsis parvispinus*, (b) *L. birchii*, (c) *L. tatei*, and (d) *L. paratatei*. Only populations for which we had at least four individuals are included; the only exception is *L. birchii* from Western Australia (locality 1) for which we only had three individuals. Population numbers correspond to numbers in Fig. 1 and Table 1. Darker shades of blue correspond to higher Φ_ST_ values. Please note that the “color scale bar” is not identical for all diagrams. Pairwise Φ_ST_ values significant at the 0.05 level are marked with an asterisk.

Analyses of COI and ITS2 show a clear subdivision of *L. parvispinus* into two geographically isolated genetic lineages (Figs. 2a and 3). One lineage occurs in New South Wales, southern Queensland, and Victoria in the catchments of the Murray–Darling Basin and the Snowy River (COI haplotypes Lp01–Lp55; Fig. 1; Tables 2 and S2; this group was referred to as *L. parvispinus*“Paroo” by Schwentner et al. 2011), the other in central Queensland in the upper catchment of the Cooper Creek (haplotypes Lp56–Lp74; this group was referred to as *L. parvispinus*“Buchanan” by Schwentner et al. 2011). This finding was supported in the AMOVA analysis by the high *F*_CT_ value of 0.795 between these lineages, with most variation occurring between them (Table 3). Pairwise Φ_ST_ values between populations of these lineages were all ≥0.79 (Fig. 4a; nearly all were significant at the 0.05 level) and the Mantel test revealed a highly significant correlation between Φ_ST_ and geographic distance, indicating isolation-by-distance (Table 2) at this geographic level. However, the SAMOVA supported a split into three groups (*F*_CT_ = 0.80): “Buchanan,”“Paroo,” and the population furthest to the east of the Murray–Darling Basin (Meandarra, locality 72 in Fig. 1; Table 1).

**Table 3:** Results of the AMOVA analyses of all four *Limnadopsis* species and of the main genetic lineages within species for cytochrome c oxidase subunit I

	*F*_SC_ (populations to groups)	*F*_ST_ (populations to whole)	*F*_CT_ (groups to whole)	Variation among groups (%)	Variation among populations within groups (%)	Variation within populations (%)
*L. parvispinus* (all, five groups = subcatchments)	0.144**	0.766**	0.727**	72.65	3.94	23.41
*L. parvispinus* (all, two groups = main basins)	0.215**	0.839**	0.795**	79.47	4.41	16.12
*L. parvispinus*“Paroo” (four groups = subcatchments)	0.101**	0.269**	0.187**	18.75	8.18	73.07
*L. birchii* (all, seven groups = subcatchments)^+^	0.110**	0.705**	0.669**	66.86	3.64	29.50
*L. birchii* (all, three groups = main basins)^+^	0.162**	0.842**	0.812**	81.15	3.06	15.79
*L. birchii*“Paroo” (five groups = subcatchments)	0.081**	0.143	0.068*	6.79	7.53	85.68
*L. tatei* (all, three groups = main basins)	−0.007	−0.050	−0.043	−4.27	−0.70	104.97
*L. paratatei* (all, two main basins)	0.245**	0.153**	−0.122	−12.20	27.46	84.74

Only populations with at least four sequences were included in the analyses. Populations correspond to single pools. Groups combine populations either on the level of main basins or subcatchments. *Significant (*P*≤ 0.05), **highly significant (*P*≤ 0.01),^+^the Western Australian population was included though only three sequences were available.

The subdivisions among populations do not correlate to subcatchments within basins. Grouping all the populations according to the five subcatchments (four within the Murray–Darling Basin, one in the Lake Eyre Basin; Fig. 1) resulted in a lower but still high *F*_CT_ value (0.727), though this is largely due to the genetic differences between the two main lineages. A similar AMOVA including only the four subcatchments of the Murray–Darling Basin (an area that corresponds to the geographic distribution of the “Paroo” lineage) resulted in an *F*_CT_ value of only 0.187 (deemed 73% of the genetic variation to occur within single populations) (Table 3). The SAMOVA also failed to support a subdivision according to subcatchments for the “Paroo” lineage. The highest *F*_CT_ value (0.588) was obtained when the Meandarra population (locality 72) was grouped separately from all other populations and did not result any further groupings within the “Paroo” lineage. The genetic distinction of the Meandarra population was further strengthened by high Φ_ST_ values (≥0.52, nearly all significant; Fig. 4a), whereas all other “Paroo” populations generated smaller, mostly nonsignificant pairwise Φ_ST_ values (Fig. 4a), indicating low or no genetic differentiation. The Mantel test did not support isolation-by-distance within the “Paroo” lineage (Table 2).

Within the “Buchanan” lineage, rather than resulting in a consistent grouping of populations, the *F*_CT_ value of the SAMOVA analyses increased with the number of proposed groups until all the populations were assigned to separate groups. Pairwise Φ_ST_ values were generally greater than within the “Paroo” lineage (Fig. 4a) and the Mantel test revealed a significant correlation between Φ_ST_ and geographic distance, indicating isolation-by-distance. It should be noted that most populations of this lineage are separated by less than 3 km; Lake Galilee (locality 10) is the furthest away from the other populations at a distance of about 100 km. Geographic distances within the “Paroo” populations are much larger (up to 1000 km; Fig. 1), although several populations were geographically close to each other.

On the basis of the COI dataset, *L. birchii* is structured into three main geographically separated lineages (Fig. 2b; Table S3): Western Australia (locality 1; haplotype Lb05), central Queensland (upper catchment of the Cooper Creek, localities 2–12; haplotypes Lb01–Lb4), and South Australia, south Queensland, and New South Wales (Murray–Darling Basin, Bulloo River, and Lake Frome drainage system, localities 13–70; haplotypes Lb6–Lb37). The latter two lineages have a similar distribution to that of the two *L. parvispinus* lineages. A single individual originating from the “Paroo” area (locality 33) featured a haplotype belonging to the *L. birchii*“Buchanan” lineage (Lb03). We double-checked this remarkable finding (repeated DNA isolation, PCR amplification, and sequencing) and can exclude contamination. The Western Australian lineage and *L. birchii*“Buchanan” are separated by 10 and six mutational steps, respectively, from *L. birchii*“Paroo,” showing that the separation is not recent (i.e., anthropogenic). The ITS2 haplotype network did not reveal any genetic structure in correlation to the geographic origin of the specimens (Fig. 3). On the contrary, a single haplotype occurred throughout the distributional range, with only a few closely related haplotypes uncovered in addition.

The AMOVA and SAMOVA for the COI dataset supported this subdivision into three main lineages (*F*_CT_ = 0.812); grouping the populations according to subcatchments resulted in a lower *F*_CT_ value (0.669). Gene flow among populations of these three lineages (or geographic areas) is restricted, as indicated by pairwise Φ_ST_ values of ≥0.80 (Fig. 4b; all significant at the 0.05 level) between their populations (due to the individual sharing a haplotype with the *L. birchii*“Buchanan” lineage, locality 33 had reduced pairwise Φ_ST_ values) and a significant correlation between Φ_ST_ and geographic distance in the Mantel test (Table 2). No differentiation exists among the *L. birchii*“Paroo” populations, as indicated by generally low and nonsignificant Φ_ST_ values (Fig. 4b) and a lack of isolation-by-distance (Table 2). Within *L. birchii*“Buchanan,” only locality 8 demonstrated high and significant pairwise Φ_ST_ values, resulting in a significant correlation between Φ_ST_ and geographic distance (Table 2).

Neither COI nor ITS2 revealed any genetic or spatial structure in either *L. tatei* or *L. paratatei* (Figs. 2c, d and 3; Tables S4 and S5) in central and eastern Australia, with identical haplotypes occurring throughout this area. However, the Western Australian specimen (P.87101) of *L. tatei* is distinct from all other specimens of this species in both genetic markers. For both species, the AMOVA deemed virtually all genetic variation to occur within populations (Table 3) and the SAMOVA failed to result in any grouping with a significant *F*_CT_ value. Φ_ST_ values were generally low (Fig. 4c and d) and not significant at the 0.05 level, and were not correlated to geographic distance (Table 2).

### Demographic history

**Figure 5:**
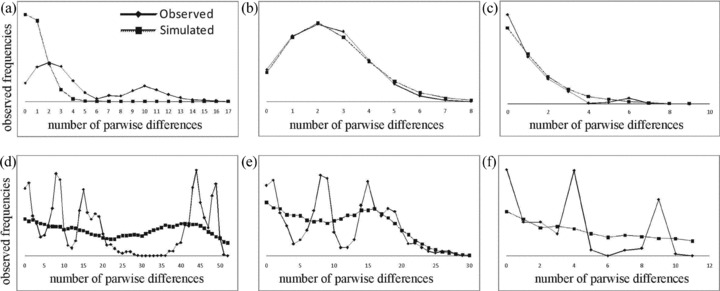
Mismatch distributions of cytochrome c oxidase subunit I under a sudden expansion model for all four *Limnadopsis* species and the main lineages within them. (a) *Limnadopsis birchii*, (b) *L. birchii*“Paroo,” (c) *L. tatei*, (d) *L. parvispinus*, (e) *L. parvispinus*“Paroo,” and (f) *L. paratatei*.

The patterns of demographic history that can be inferred from the COI dataset are somewhat ambiguous. Population expansion is supported for *L. parvispinus*“Paroo,”*L. tatei*, *L. birchii*, *L. birchii*“Paroo,” and *L. birchii*“Buchanan” by Fu's *F*_s_, but only for *L. tatei*, *L. birchii*, and *L. birchii*“Paroo” by Tajima's *D* (Table 2). The other species and lineages exhibit either nonsignificant negative or even positive values; the latter being an indication of populations that have not undergone expansion (e.g., *L. paratatei*; Table 2). In the mismatch distribution, sudden demographic expansion was not consistently refused for any species or lineage going by the SSD and Hri values (Table 2), and only one of the indices did refuse the sudden expansion model for *L. birchii* and *L. paratatei*. This is unexpected, as the mismatch distributions for *L. parvispinus*, *L. parvispinus*“Paroo,”*L. paratatei*, and *L. birchii* are clearly multimodal (Fig. 5), which is typical of stable, nonexpanding populations. *Limnadopsis parvispinus* and *L. parvispinus*“Paroo” in particular show mismatch distributions at odds with expanding populations.

### Genetic distances and divergence times

The COI genetic distances observed (uncorrected *p*-distances) were greatest in *L. parvispinus*, at up to 9.9% between the “Paroo” and “Buchanan” lineages and 5.6% and 3.3%, respectively, within these lineages (Table 4). The genetic distances within the other three species were much lower, at up to 3.1% in *L. birchii*, 1.7% in *L. paratatei*, and 1.6% in *L. tatei* (Table 4). ITS2 genetic distances are lower than COI distances (Table 5), but it should be borne in mind that indels are not accounted for in these calculations. Within *L. parvispinus*, the largest observed ITS2 distance was 4.0%, and within its two main lineages just 0.5% and 1.5%, respectively. The other three species have intraspecific ITS2 distances of up to 0.9%. Noteworthy is the low interspecific genetic distance of just 0.5–1.6% between *L. tatei* and *L. birchii*, which overlaps with the intraspecific distances observed within these two species (Table 5). However, both species are characterized by several apomorphic characters, which clearly distinguish them from one another. The largest ITS2 distances within *L. tatei* occurred between the Western Australian specimen (P.87801) and specimens from central and eastern Australia.

**Table 4:** Cytochrome c oxidase subunit I genetic distances within species and within and between main genetic lineages

	Uncorrected *p*-distance in percentage	K2P distance in percentage	Divergence time in mya (rate 1.4%)	Divergence time in mya (rate 2.3%)
*L. parvispinus*	0.0–9.9	0.0–10.8	0.0–7.1	0.0–4.7
*L. parvispinus* “Paroo”	0.0–5.6	0.0–5.9	0.0–4.2	0.0–2.6
*L. parvispinus* “Buchanan”	0.0–3.3	0.0–3.4	0.0–2.4	0.0–1.5
*L. parvispinus* “Paroo” versus “Buchanan”	6.6–9.9	7.2–10.8	5.1–7.1	3.1–4.7
*L. birchii*	0.0–3.1	0.0–3.2	0.0–2.3	0.0–1.4
*L. birchii* “Paroo”	0.0–1.3	0.0–1.4	0.0–1.0	0.0–0.6
*L. birchii* “Buchanan”	0.0–0.4	0.0–0.4	0.0–0.3	0.0–0.2
*L. birchii* “Western Australia”	0.0	0.0	-	-
*L. birchii* “Paroo” versus “Buchanan”	1.3–2.7	1.4–2.8	1.0–2.0	0.6–1.2
*L. birchii* “Paroo” versus “Western Australia”	1.9–3.1	2.0–3.2	1.4–2.3	0.9–1.4
*L. birchii* “Buchanan” versus “Western Australia”	2.9–3.1	3.0–3.2	2.1–2.3	1.3–1.4
*L. tatei*	0.0–1.5	0.0–1.6	0.0–1.1	0.0–0.7
*L. tatei* without Western Australia	0.0–0.8	0.0–0.8	0.0–0.6	0.0–0.3
*L. tatei* Western Australia versus all others	1.0–1.5	1.0–1.6	0.7–1.1	0.4–0.7
*L. paratatei*	0.0–1.7	0.0–1.8	0.0–1.3	0.0–0.8

Divergence times in million years ago (mya) were calculated on the basis of the K2P distance, applying the divergence rates of 1.4% and 2.3% per million years, respectively. K2P, Kimura-2-Parameter.

**Table 5:** Internal transcribed spacer 2 genetic distances within and between species

	*L. parvispinus* “Paroo”	*L. parvispinus* “Buchanan”	*L. birchii*	*L. tatei*	*L. paratatei*
*L. parvispinus* “Paroo”	0.0–0.5	1.9–4.0	8.2–10.3	8.4–10.3	8.9–10.1
*L. parvispinus* “Buchanan”		0.0–1.5	8.1–10.1	8.4–10.3	8.7–10.2
*L. birchii*			0.0–0.8	0.5–1.6	1.8–2.4
*L. tatei*				0.0–0.9	2.1–2.9
*L. paratatei*					0.0–0.2

All distances are uncorrected *p*-distances. Indels were not included in distance calculations.

**Figure 6:**
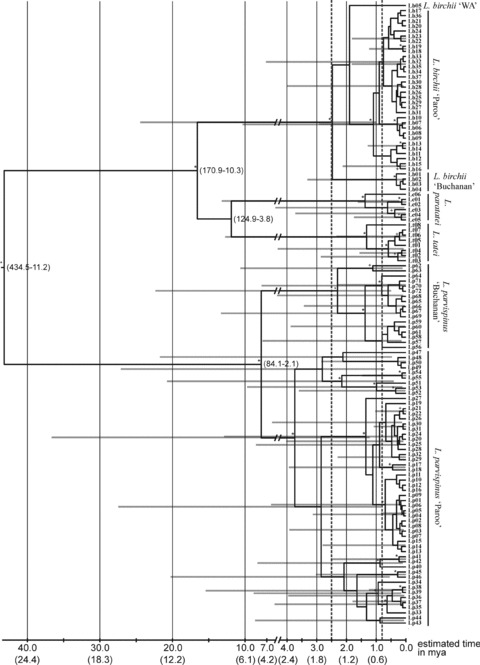
Phylogenetic tree of all cytochrome c oxidase subunit I haplotypes of all four *Limnadopsis* species with uncorrelated relaxed molecular clock divergence time estimates. Divergence times were estimated in substitutions per site and have been transformed into time in million years on the basis of the divergence rate of 1.4% per million years. Time estimates based on the 2.3% per million years divergence rate are given in brackets below. The gray horizontal bars represent the 95% confidence interval for the respective nodes. A few nodes had confidence intervals exceeding the time frame shown, these confidence intervals are given at the respective nodes. The vertical dotted lines mark the 2.5 (beginning of Pleistocene) and 0.8 (mid-Pleistocene) million years time horizons, estimated on the basis of the 1.4% per million years divergence rate. Note the change in the scale bar after about 4 mya. +, posterior probability >0.90; *, posterior probability >0.95.

All time estimates are based on the COI dataset and a divergence rate of 1.4% per million years (Fig. 6 and Table 4), as this appears most appropriate for branchiopods. A higher divergence rate (e.g., 2.3%) naturally leads to younger divergence time estimates, as shown in Figure 6 and Table 4. Divergence time estimates based on the relaxed molecular clock are not very precise anyhow, as evidenced by large (95%) confidence intervals, which impede accurate divergence time estimates (Fig. 6). All divergence time estimates should be treated with caution, though the sequence of divergence events and their timing relative to each other can be regarded as more reliable. The ranges reported only reflect direct estimates based on genetic distances and those of the respective node in the dated tree. The actual ranges of the latter are shown in Figure 6.

The majority of haplotypes in all four species originated at some point within the last 0.5–0.8 million years, according to the relaxed molecular clock estimate. Deeper intraspecific splits occurred largely during the last 2.0–2.5 million years (divergences of that age have 95% confidence intervals of approximately 1–25mya). However, the oldest intraspecific split within *L. parvispinus*—that between the “Buchanan” and “Paroo” populations—dates to around 5–7.5 mya. The “Paroo” lineage diverged further around 4 mya, while the divergence within the “Buchanan” lineages occurred later (about 2.0–2.4 mya). Within *L. birchii*, the three main lineages diverged about 1–2.5 mya. The order in which they did so is ambiguous, going by genetic distances (Table 4) and the lack of nodal support in the dated tree (Fig. 6), and it is possible that they diverged simultaneously. The oldest divergences within *L. tatei* and *L. paratatei* date to around 1.3 mya each. Interspecific divergences date to around 12 mya or earlier. The oldest split (between *L. parvispinus* and all other species) dates to about 43 mya, with the lower bound of the 95% confidence interval extending to around 11 mya.

## Discussion

### Large-scale phylogeographic patterns

A comparison of the phylogeographic patterns revealed by the COI dataset in the four *Limnadopsis* species revealed overall congruencies in the spatial distribution of genetic lineages. The eastern Australian populations of *L. parvispinus* and *L. birchii* are differentiated into lineages inhabiting central Queensland (upper catchment of the Cooper, “Buchanan”) and lineages inhabiting the Murray–Darling Basin (Paroo). This finding ties in with the results of studies of river-inhabiting species such as fish, crayfish, and mollusks (Carini and Hughes 2004; Hughes et al. 2004; Carini et al. 2006; Huey et al. 2006; Hughes and Hillyer 2006; Faulks et al. 2010a,b), whose populations are generally structured according to main drainage systems or basins (Hughes et al. 2009). As dispersal of these species depends on their habitats connecting up during floods, they are not able to cross between main river basins. The divergence of lineages inhabiting the Murray–Darling and Lake Eyre Basins has been dated to between 0.15 and 1.5 mya (Carini et al. 2006; Hughes and Hillyer 2006). This is similar to the estimated divergence time of the respective *L. birchii* populations, but more recent than the estimated divergence of the respective populations of *L. parvispinus* (about 5–7 mya).

However, the genetic differentiation (seemingly between drainage systems) is only evident along the north–south axis in eastern Australia. The populations of *L. birchii*, *L. tatei*, and *L. paratatei*, which inhabit the Bulloo catchment and southern (Lake Frome) and westerns (Finke River) areas of the Lake Eyre Basin (*L. parvispinus* has not been found in these areas), are genetically very similar or even identical to the populations of the Murray–Darling Basin. Similarly, no genetic subdivision corresponding to the subcatchments of the Murray–Darling Basin was revealed in any *Limnadopsis* species. The high number of haplotypes exhibiting wide spatial distribution indicates recent and numerous independent dispersal and gene flow events over large geographic distances (distances >1000 km, covering an area of >800,000 km^2^) and across drainage systems borders. This cannot be a result of actual drainage connectivity between the river basins concerned. We suggest that the lack of genetic differentiation over large geographic distances is down to a mode of dispersal, which is independent of the direct connection of habitats and the connection of river systems (see section Potential Dispersal Vectors). As genetic differentiation across drainage systems is low or nonexistent along the east–west axis (at least up to central Australia), also the genetic differentiation of the central Queensland populations (Buchanan) may be due to other dispersal-limiting factors than the actual drainage division (see section Potential Dispersal Vectors). Populations in the far west of Australia (e.g., *L. birchii* and *L. tatei* from Western Australia) appear to be genetically distinct from other populations, probably to the same extent as observed between *L. birchii* “Paroo” and “Buchanan,” but the limited number of individuals studied does not allow any clear conclusions to be drawn.

The ITS2 dataset did not indicate any geographic subdivision other than that between *L. parvispinus* “Paroo” and “Buchanan.” However, the very low overall intraspecific diversity, which has been reported for other crustaceans as well (Schön et al. 2010), suggests a low evolutionary rate for ITS2. This, rather than gene flow, may have caused the lack of genetic structure observed among far-flung populations of *L. birchii*.

### Correlation between species divergence and Australia's climate

*Limnadopsis* species are not dependent on aridity per se, but rather on the availability of temporary water bodies. Suitable water bodies may have been present outside the areas dominated by rainforest and are likely to have been widespread in the late Eocene and Oligocene (Hill 2004; Martin 2006), the period when the earliest divergences within *Limnadopsis* may have occurred.

According to the dated tree of the relaxed molecular clock analysis, the diversification of many basal intraspecific lineages within all four studied *Limnadopsis* species appears to have occurred within the 2.5 million years since the beginning of the Pleistocene, with most of the present day lineages appearing since the mid-Pleistocene (∼0.8 mya). However, due to the inherent uncertainties in the dating of diversification events, the actual diversification may have occurred much earlier. Nevertheless, the inferred time periods are associated with important changes in climate and thus vegetation. Glacial cycling started at the beginning of the Pleistocene and changed in the mid-Pleistocene to cycles of longer periodicity and greater amplitude. The alternation of wet interglacial and dry glacial periods was overlaid by an overall trend toward aridification and an expansion of the arid zone in Australia (Martin 2006; Byrne et al. 2008). The availability of temporary water bodies suitable for *Limnadopsis* species may have increased during dry periods. Prolonged wet periods with extensive and/or recurring rainfall would have resulted in large, (nearly) permanent water bodies and reduced the number of adequate temporary habitats. *Limnadopsis* and other branchiopod species are therefore likely to have benefited from the progressing aridification of Australia, with the increase in available habitats resulting in expanding populations. All four species studied provide some indication of recent expansion events. Some deviations (such as the clear multimodal mismatch distribution curve in *L. parvispinus* “Paroo”) may be explained by the retention of divergent “old” lineages and the existence of divergent haplotypes in refugial areas (see section Temporal Dispersal and the Role of “Old” Haplotypes; Hewitt 1996). Several sublineages in all species, however, display clear star-like haplotype distributions with one or a few widely distributed central haplotypes, a pattern typical of recent expansions. Examples are haplotypes Lp01–Lp32 in *L. parvispinus* “Paroo” (two star-like patterns with Lp05 and Lp20 in the centers), *L. birchii* “Buchanan,” (Lb03 central) and *L. birchii* “Paroo” (Lb08, Lb12, Lb20, and Lb26 are potential centers). The combination of low genetic diversity and wide haplotype distribution in *L. tatei* and *L. paratatei* also indicates recent expansions of these species across central and southeastern Australia and explains why no clear pattern of genetic structuring is seen over these large areas of arid zone Australia. Byrne (2008) and Byrne et al. (2008) reviewed the genetic patterns of species inhabiting the arid zone of Australia and concluded that most species were geographically structured due to the existence of multiple localized refugia and a subsequent lack of extensive migration or expansion. Only some vagile species (e.g., the King Brown snake, Kuch et al. 2005, or the Spiny-cheeked Honeyeater, Joseph and Wilke 2007) show patterns of expansion across arid zone Australia similar to those observed in *Limnadopsis* (Byrne et al. 2008).

For *L. parvispinus* and *L. birchii*, the Murray–Darling Basin and to a lesser extent central Queensland appear to represent important long-term refugial areas (the Bulloo and the southern Lake Eyre Basin may have also been important for *L. birchii*). This is indicated by the high overall genetic diversity and deep haplotype divergence observed in these areas. According to the estimated divergence times of these haplotypes, *L. parvispinus* “Paroo” may have inhabited the Murray–Darling Basin continuously for the last 4 million years and *L. birchii* “Paroo” for the last 1 million years. Consequently, suitable habitats would have persisted in this area in both dry glacial and wet interglacial periods. Later immigration from other areas is another possibility, though it is unlikely that such a genetically rich and divergent assembly of haplotypes would be the result of immigration only, especially as no other areas, which may have acted as source populations, are known to be inhabited by *L. parvispinus* today (Timms 2009), and no potential source population was found for *L. birchii* throughout the studied area. The number of sites sampled in Western Australia is too low to make valid assumptions about their role as refugial areas for *L. birchii*. However, the split from the eastern populations has been dated to have occurred earlier than 2 mya, suggesting long-term refugia in Western Australia as well.

*Limnadopsis tatei* and *L. paratatei* exhibit low genetic diversity and shallow haplotype divergence throughout the areas studied. No obvious refugial area can, therefore, be pinpointed. If the species inhabited the areas in question continuously, they may have been restricted to very few sites, resulting in an overall loss of genetic diversity. This scenario implies that the Murray–Darling Basin did not provide enough suitable habitats for these two species to retain much genetic diversity through the Pleistocene or even to persist at all. Alternatively, the refugial areas of greater genetic diversity are located outside the study area (e.g., further to the west or north), in which case the two species would have expanded into central and eastern Australia only recently. Under this scenario, either a lack of suitable habitats during the Pleistocene or factors limiting the dispersal ability of these species may have prevented them from inhabiting the Murray–Darling Basin earlier. Even today neither species is as prevalent in this area as *L. parvispinus* or *L. birchii* (Timms and Richter 2002; Timms 2009; the distinction between *L. tatei* and *L. paratatei* was not made therein). Differences in their distribution and occurrence may reflect distinct habitat preferences that vary between species (Timms and Richter 2002). A better understanding of today's habitats and the habitat preferences of the species in question may help us to understand the historical composition of temporary water bodies in arid zone Australia.

### Potential dispersal vectors

Branchiopods and other freshwater invertebrates with resting eggs (or other resting stages) are generally assumed to have a high potential for dispersal. Many of these species are among the first colonizers of new habitats (reviewed in De Meester et al. 2002), and dispersal via wind, birds, or other animals should theoretically facilitate unrestricted gene flow. However, a range of studies has revealed pronounced phylogeographic structures in the spatial distribution of genetic lineages, even on small spatial scales (reviewed in De Meester et al. 2002). Populations separated by a few to a few hundred kilometers (e.g., Vanoverbeke and De Meester 1997; Brendonck et al. 2000; Hulsmans et al. 2007; Thielsch et al. 2009) were genetically differentiated, displaying high *F*_ST_ or Φ_ST_ values. Surprisingly, the four *Limnadopsis* species studied do not fit this pattern of genetic differentiation observed in other branchiopods. Apart from the differentiation into the “Buchanan,”“Paroo,” and Western Australian lineages, they show little sign of genetic differentiation on either the local or the broader scale. The numerous populations studied across the Murray–Darling, the Bulloo, and southern and western Lake Eyre Basin in particular appear to show little to no genetic differentiation, yet, in *L. birchii* and *L. parvispinus* at least, genetic diversity is high. The lack of genetic structure in *L. tatei* and *L. paratatei* appears to be due to their overall low genetic diversity. Such low genetic differentiation over larger spatial scales has been observed in a few branchiopod species as well, (Naihong et al. 2000; Cesari et al. 2007; Muñoz et al. 2010), indicating expansion events that were too recent for genetic diversification and differentiation to have been established.

Nevertheless, the lack of genetic structure observed in *L. parvispinus* and *L. birchii* and probably also of *L. tatei* and *L. paratatei*, contradicts the monopolization hypothesis (De Meester et al. 2002), which explains the often observed incongruity of high dispersal potential coinciding with low gene flow in terms of persisting founder effects coupled with local adaptations and the numerical predominance of the local population (De Meester et al. 2002). The latter factor is intensified by resting egg banks that increase the size of the population and act as a buffer against newly invading genetic lineages (Boileau et al. 1992; De Meester et al. 2002). The patterns observed here for *Limnadopsis* rather fit the original idea that branchiopods are good dispersers with high gene flow. This is most likely explained by factors that promote high dispersal efficiency in these parts of Australia, as there is no indication that the *Limnadopsis* species themselves are better adapted to dispersal or gene flow than other branchiopod species. Of course, the lack of genetic differentiation may be furthered by other factors such as the persistence of founding haplotypes and/or a substitution rate, which is too low to establish genetic diversification and differentiation. The genetic diversity observed within *L. parvispinus* and *L. birchii*, however, suggests that this is not the case. A number of haplotypes have wide distribution ranges, indicating that several independent dispersal events took place. The populations may not be in full panmixia, but occasional dispersal and gene flow appears plausible.

Dispersal of the four *Limnadopsis* species among drainage systems is unlikely to be correlated to flood events, as floods of the magnitude required to connect drainage systems are extremely rare, if they occur at all. It has long been known that birds can passively disperse freshwater organisms (Darwin 1859) and that many resting eggs are able to survive the gut passage of birds (Proctor 1964; Green et al. 2005). Green et al. (2008) studied the number and frequency of resting eggs in the feces of the bird species Grey Teal (*Anas gracilis*), Eurasian Coot (*Fulica atra*), Black Swan (*Cygnus atrus*), and Australian Pelican (*Pelicanus conspicillatus*) feeding in the Macquarie Marshes (located within the Murray–Darling Basin). “Large branchiopod” resting eggs were found in 30% of the fecal samples and were retrieved from all bird species studied. The highest percentage (50%) of “large branchiopod” egg-containing fecal samples came from the Grey Teal, and Australian Pelican feces contained the largest absolute number of resting eggs. As all of these bird species are highly nomadic, they have the capacity to disperse freshwater invertebrates over large areas (Green et al. 2008). Migration distances of up to 300 km in one day have been recorded for Grey Teal (Roshier et al. 2006, 2008), and long-term monitoring has shown large-scale migration movements covering large areas of the Lake Eyre and Murray–Darling Basins (Roshier et al. 2008). Computer models based on the availability of wetland habitats in arid Australia over an 11-year period (1986–1997) showed overall connectivity in the wetland habitats for migrating water birds (Roshier et al. 2001). If water birds are assumed to be able to migrate up to 200 km between wetlands, large areas of the Lake Eyre and Murray–Darling Basins are interconnected via migratory corridors (Roshier et al. 2001). The connectivity of the temporary wetland habitats coupled with the huge numbers of migratory water birds inhabiting these wetlands (1 million water birds were estimated to inhabit the lower Cooper catchment in 1990; Kingsford et al. 1999, and several hundred thousand the Paroo catchment; Kingsford and Porter 1999) and their frequent ingestion of resting eggs may well explain the low level of population differentiation observed in these areas for all four *Limnadopsis* species.

Interestingly, the areas that harbored the most divergent populations, for example, the northern Cooper catchment (*L. parvispinus* and *L. birchii* “Buchanan”) and Meandarra in the east of the Murray–Darling Basin (locality 72), were not interconnected with the other wetland habitats of the Lake Eyre and Murray–Darling Basins by migratory corridors suitable for water birds (Roshier et al. 2001, 2008). Therefore, dispersal events to and from these areas via birds would be infrequent. The presence of a single “Buchanan” haplotype in the “Paroo” population of *L. birchii* highlights the fact that though rare, dispersal events do occur between these areas but they may be too infrequent to permit effective gene flow. The populations inhabiting pools close to Lake Buchanan and Lake Galilee also showed higher pairwise Φ_ST_ values than “Paroo” populations, although the pools in question are close together. The lower overall connectivity of wetlands in this area (Roshier et al. 2001) may reduce the level of resting egg dispersal and thus results in low effective gene flow due to founder effects and the numerical advantage of the local population (monopolization hypothesis; De Meester et al. 2002).

Wind may also play an important role in the dispersal of resting eggs (Cáceres and Soluk 2002). Studies have shown that even minor wind speeds can facilitate windborne dispersal of branchiopod eggs (Graham and Wirth 2008; Vanschoenwinkel et al. 2008). Dust storms are a common phenomenon in arid Australia and can affect large areas (McTainsh et al. 2005), traveling from the center of Australia to the east coast. The dominating wind direction in arid Australia is from west to east (http://www.bom.gov.au/climate/averages/wind/IDCJCM0021_wind_roses.shtml). Wind-mediated gene flow should thus be unidirectional from western areas to eastern areas. If wind was the main force behind the dispersal of resting eggs, haplotypes originating in eastern Australia would not disperse to the west. However, the haplotype distributions in the present study did not reveal such unidirectional dispersal, so it seems that while wind may disperse resting eggs over large distances at times, the effect of migrating birds is likely to be stronger.

Floods may also disperse resting eggs on a local scale by connecting neighboring pools after exceptionally strong rain falls. However, the effect of flooding is probably restricted to neighboring pools within subcatchments. If floods were the main dispersal agent, populations should show some genetic structuring according to subcatchments and main basins, which was not the case.

In summary, the congruent pattern of virtually no genetic differentiation over large areas (Murray–Darling, Bulloo, and south and west Lake Eyre Basins, approximately 800,000 km^2^), a phenomenon that has not been observed to this extent in any other branchiopod species, may be attributable to the dispersal capacity of the abundant, vagile migratory water birds that inhabit east and central Australia during wet periods. Wind and local floods may further facilitate the dispersal of resting eggs locally. The number of resting eggs being dispersed on a regular basis may be large enough to overrule founder effects and the buffering effects of resting egg banks. This may also explain why *Limnadopsis* exhibits patterns of range expansion similar to those of vagile arid zone taxa like the King Brown snake or the Spiny-cheeked Honeyeater (see section Correlation between Species Divergence and Australia's Climate).

### Temporal dispersal and the role of “old” haplotypes

Resting egg banks play an integral role in shaping the genetic structure of populations. Not all eggs hatch in the wet period after they are laid but over a range of wet periods (Brendonck 1996). This bet-hedging strategy is thought to spread the risk of reproductive failure over several generations (Philippi and Seger 1989; Brendonck 1996), but it also has important evolutionary implications. Haplotypes, which may not have been present in the preceding active adult generation, may “reappear” from eggs laid several generations ago. This process has been termed temporal dispersal, as it resembles “normal” dispersal among populations (Hairston and Kearns 2002). The eggs, which hatch generations after they were laid (and their haplotypes), have presumably not been affected by the evolutionary constraints that acted on previous adult generations. Haplotypes, which would otherwise have been extinct, can thus be reintroduced into the active adult population from the resting egg bank, slowing down the extinction of haplotypes caused by genetic drift and allowing populations to retain “old” haplotypes (Ellner and Hairston 1994; Hairston and Kearns 2002). The outcome is likely to be increased genetic diversity and genetically divergent haplotypes, especially in old populations. Although some branchiopod resting eggs have been shown still to be viable after 125 years (reviewed in De Stasio 2007), a delay in hatching of just a few generations is thought to influence the maintenance of genetic diversity.

This effect may be evident in the *L. parvispinus* populations. Both the “Paroo” and “Buchanan” populations have existed for long periods of time, and the genetic diversity (in terms of haplotype and nucleotide diversity) in both lineages is high. The high nucleotide diversity reflects the long differentiation history of the haplotypes, which is underlined by uncorrected *p*-distances of up to 5.6% in the “Paroo” lineage. The “Paroo” lineage in particular features a number of highly divergent haplotypes (Lp33–Lp55) that are separated by numerous mutational steps from all other haplotypes and found only in one or a few individuals. These divergent haplotypes might represent remnants of haplotype lineages, which are largely extinct. These “old” haplotypes may have been retained in the populations through the preserving effect of the resting egg bank.

## Supporting Information

**Table S1.** Detailed information on each specimen.

**Table S2.** Distribution of haplotypes of *L.parvispinus* for all localities.

**Table S3.** Distribution of haplotypes of *L. birchii* for all localities.

**Table S4.** Distribution of haplotypes of *L. tatei* for all localities.

**Table S5.** Distribution of haplotypes of *L.paratatei* for all localities.
