# Supplementary material for: Flying with the birds? Recent large-area dispersal of four Australian Limnadopsis species (Crustacea: Branchiopoda: Spinicaudata)
Source: Ecol Evol. 2012 Jul;2(7):1605–26. doi: 10.1002/ece3.265 (PMC3434933; doi:10.1002/ece3.265)
Supplement: Supplementary file 1 [file ece30002-1605-SD1.doc]

Supplement 1: Detailed information on each specimen. For each specimen the locality number (corresponds to Fig. 1, Table 1), the registration number at the Australian Museum (AM) and the GenBank accession numbers for COI and ITS2 are given. *= private collection BVT

| Locality | Species | AM reg. number | Genbank COI | Genbank ITS2 |
| --- | --- | --- | --- | --- |
| 1 | *L. birchii* | P.85108 | JN698392 | JQ624914 |
| 1 | *L. birchii* | P.85157 | JN698441 | JQ624915 |
| 1 | *L. birchii* | P.85158 | JN698442 | JQ624916 |
| 10 | *L. birchii* | P.85113 | JN698397 | JQ624918 |
| 10 | *L. birchii* | P.85114 | JN698398 |  |
| 10 | *L. birchii* | P.86341 | JN698530 |  |
| 10 | *L. birchii* | P.86342 | JN698531 |  |
| 10 | *L. birchii* | P.86343 | JN698532 |  |
| 11 | *L. birchii* | P.85129 | JN698413 |  |
| 11 | *L. birchii* | P.85130 | JN698414 |  |
| 11 | *L. birchii* | P.86274 | JN698463 |  |
| 11 | *L. birchii* | P.86275 | JN698464 | JQ624919 |
| 11 | *L. birchii* | P.86276 | JN698465 |  |
| 12 | *L. birchii* | P.85102 | JN698386 |  |
| 13 | *L. birchii* | P.85151 | JN698435 |  |
| 13 | *L. birchii* | P.85152 | JN698436 | JQ624917 |
| 13 | *L. birchii* | P.86338 | JN698527 |  |
| 13 | *L. birchii* | P.86339 | JN698528 |  |
| 13 | *L. birchii* | P.86340 | JN698529 |  |
| 14 | *L. birchii* | P.86290 | JN698479 |  |
| 14 | *L. birchii* | P.86291 | JN698480 |  |
| 14 | *L. birchii* | P.86292 | JN698481 | JQ624929 |
| 14 | *L. birchii* | P.86293 | JN698482 | JQ624929 |
| 14 | *L. birchii* | P.86294 | JN698483 | JQ624931 |
| 14 | *L. birchii* | P.86295 | JN698484 |  |
| 14 | *L. birchii* | P.86296 | JN698485 |  |
| 14 | *L. birchii* | P.86297 | JN698486 |  |
| 16 | *L. birchii* | P.86282 | JN698471 |  |
| 16 | *L. birchii* | P.86283 | JN698472 |  |
| 16 | *L. birchii* | P.86284 | JN698473 |  |
| 17 | *L. birchii* | P.86285 | JN698474 |  |
| 17 | *L. birchii* | P.86286 | JN698475 |  |
| 17 | *L. birchii* | P.86287 | JN698476 |  |
| 17 | *L. birchii* | P.86288 | JN698477 |  |
| 17 | *L. birchii* | P.86289 | JN698478 |  |
| 18 | *L. birchii* | P.86298 | JN698487 |  |
| 18 | *L. birchii* | P.86299 | JN698488 |  |
| 18 | *L. birchii* | P.86300 | JN698489 |  |
| 18 | *L. birchii* | P.86301 | JN698490 |  |
| 18 | *L. birchii* | P.86302 | JN698491 |  |
| 19 | *L. birchii* | P.86308 | JN698497 |  |
| 19 | *L. birchii* | P.86309 | JN698498 |  |
| 19 | *L. birchii* | P.86310 | JN698499 |  |
| 19 | *L. birchii* | P.86311 | JN698500 |  |
| 19 | *L. birchii* | P.86312 | JN698501 |  |
| 20 | *L. birchii* | P.86303 | JN698492 | JQ624932 |
| 20 | *L. birchii* | P.86304 | JN698493 |  |
| 20 | *L. birchii* | P.86305 | JN698494 |  |
| 20 | *L. birchii* | P.86306 | JN698495 |  |
| 20 | *L. birchii* | P.86307 | JN698496 |  |
| 24 | *L. birchii* | P.84148 | HQ717756 | JQ624924 |
| 24 | *L. birchii* | P.84149 | HQ717757 | JQ624922 |
| 24 | *L. birchii* | P.84150 | HQ717758 | JQ624926 |
| 24 | *L. birchii* | P.85160 | JN698444 |  |
| 26 | *L. birchii* | P.84151 | FJ830343 | JQ624923 |
| 27 | *L. birchii* | P.85110 | JN698394 |  |
| 27 | *L. birchii* | P.85111 | JN698395 |  |
| 27 | *L. birchii* | P.85112 | JN698396 |  |
| 28 | *L. birchii* | P.85122 | JN698406 |  |
| 29 | *L. birchii* | P.86320 | JN698509 |  |
| 29 | *L. birchii* | P.86321 | JN698510 |  |
| 31 | *L. birchii* | P.85154 | JN698438 |  |
| 31 | *L. birchii* | P.85155 | JN698439 |  |
| 32 | *L. birchii* | P.85117 | JN698401 |  |
| 33 | *L. birchii* | P.85123 | JN698407 | JQ624920 |
| 33 | *L. birchii* | P.85124 | JN698408 |  |
| 33 | *L. birchii* | P.86268 | JN698457 |  |
| 33 | *L. birchii* | P.86269 | JN698458 |  |
| 33 | *L. birchii* | P.86270 | JN698459 |  |
| 34 | *L. birchii* | P.85131 | JN698415 |  |
| 34 | *L. birchii* | P.85132 | JN698416 |  |
| 38 | *L. birchii* | P.85137 | JN698421 | JQ624921 |
| 38 | *L. birchii* | P.85138 | JN698422 |  |
| 45 | *L. birchii* | P.85116 | JN698400 |  |
| 46 | *L. birchii* | P.85120 | JN698404 |  |
| 46 | *L. birchii* | P.85121 | JN698405 |  |
| 48 | *L. birchii* | P.85109 | JN698393 |  |
| 50 | *L. birchii* | P.84147 | HQ717755 |  |
| 51 | *L. birchii* | P.86324 | JN698513 |  |
| 51 | *L. birchii* | P.86325 | JN698514 |  |
| 52 | *L. birchii* | P.86322 | JN698511 |  |
| 52 | *L. birchii* | P.86323 | JN698512 |  |
| 53 | *L. birchii* | P.85106 | JN698390 |  |
| 53 | *L. birchii* | P.85107 | JN698391 |  |
| 54 | *L. birchii* | P.85119 | JN698403 |  |
| 54 | *L. birchii* | P.85142 | JN698426 |  |
| 54 | *L. birchii* | P.86265 | JN698454 |  |
| 54 | *L. birchii* | P.86266 | JN698455 |  |
| 54 | *L. birchii* | P.86267 | JN698456 |  |
| 55 | *L. birchii* | P.85140 | JN698424 |  |
| 55 | *L. birchii* | P.85141 | JN698425 |  |
| 56 | *L. birchii* | P.85115 | JN698399 |  |
| 56 | *L. birchii* | P.85139 | JN698423 |  |
| 56 | *L. birchii* | P.86262 | JN698451 |  |
| 56 | *L. birchii* | P.86263 | JN698452 |  |
| 56 | *L. birchii* | P.86264 | JN698453 |  |
| 57 | *L. birchii* | P.86313 | JN698502 |  |
| 57 | *L. birchii* | P.86314 | JN698503 |  |
| 57 | *L. birchii* | P.86315 | JN698504 |  |
| 57 | *L. birchii* | P.86316 | JN698505 |  |
| 57 | *L. birchii* | P.86317 | JN698506 |  |
| 58 | *L. birchii* | P.86318 | JN698507 | JQ624934 |
| 58 | *L. birchii* | P.86319 | JN698508 |  |
| 59 | *L. birchii* | P.86326 | JN698515 |  |
| 59 | *L. birchii* | P.86327 | JN698516 |  |
| 59 | *L. birchii* | P.86328 | JN698517 |  |
| 59 | *L. birchii* | P.86329 | JN698518 |  |
| 60 | *L. birchii* | P.86330 | JN698519 | JQ624933 |
| 60 | *L. birchii* | P.86331 | JN698520 |  |
| 61 | *L. birchii* | P.86257 | JN698446 |  |
| 61 | *L. birchii* | P.86258 | JN698447 |  |
| 61 | *L. birchii* | P.86259 | JN698448 |  |
| 61 | *L. birchii* | P.86260 | JN698449 |  |
| 61 | *L. birchii* | P.86261 | JN698450 |  |
| 62 | *L. birchii* | P.85118 | JN698402 |  |
| 63 | *L. birchii* | P.85145 | JN698429 |  |
| 63 | *L. birchii* | P.85146 | JN698430 |  |
| 64 | *L. birchii* | P.85143 | JN698427 |  |
| 64 | *L. birchii* | P.85144 | JN698428 |  |
| 64 | *L. birchii* | P.86271 | JN698460 |  |
| 64 | *L. birchii* | P.86272 | JN698461 |  |
| 64 | *L. birchii* | P.86273 | JN698462 |  |
| 66 | *L. birchii* | P.85135 | JN698419 |  |
| 66 | *L. birchii* | P.85136 | JN698420 | JQ624927 |
| 67 | *L. birchii* | P.85147 | JN698431 |  |
| 67 | *L. birchii* | P.85148 | JN698432 |  |
| 67 | *L. birchii* | P.86335 | JN698524 |  |
| 67 | *L. birchii* | P.86336 | JN698525 |  |
| 67 | *L. birchii* | P.86337 | JN698526 |  |
| 68 | *L. birchii* | P.85149 | JN698433 |  |
| 68 | *L. birchii* | P.85150 | JN698434 |  |
| 68 | *L. birchii* | P.86332 | JN698521 |  |
| 68 | *L. birchii* | P.86333 | JN698522 |  |
| 68 | *L. birchii* | P.86334 | JN698523 |  |
| 69 | *L. birchii* | P.85127 | JN698411 | JQ624928 |
| 69 | *L. birchii* | P.85128 | JN698412 |  |
| 69 | *L. birchii* | P.86277 | JN698466 |  |
| 69 | *L. birchii* | P.86278 | JN698467 |  |
| 69 | *L. birchii* | P.86279 | JN698468 |  |
| 70 | *L. birchii* | P.85159 | JN698443 |  |
| 71 | *L. birchii* | P.85156 | JN698440 |  |
| 71 | *L. birchii* | P.86281 | JN698470 |  |
| 37a | *L. birchii* | P.85153 | JN698437 |  |
| 37a | *L. birchii* | P.86256 | JN698445 |  |
| 65a | *L. birchii* | P.85103 | JN698387 |  |
| 65a | *L. birchii* | P.85104 | JN698388 |  |
| 65a | *L. birchii* | P.86280 | JN698469 |  |
| 65b | *L. birchii* | P.85125 | JN698409 |  |
| 65b | *L. birchii* | P.85126 | JN698410 |  |
| 7a | *L. birchii* | P.85105 | JN698389 |  |
| 7a | *L. birchii* | P.85133 | JN698417 |  |
| 7a | *L. birchii* | P.85134 | JN698418 |  |
| 7a | *L. birchii* | P.87797 | JN698385 |  |
| 7b | *L. birchii* | P.84152 | HQ717759 |  |
| 17 | *L. paratatei* | P.86402 | JN698577 | JQ624937 |
| 17 | *L. paratatei* | P.86403 | JN698578 | JQ624938 |
| 17 | *L. paratatei* | P.86404 | JN698579 |  |
| 18 | *L. paratatei* | P.86405 | JN698580 |  |
| 18 | *L. paratatei* | P.86406 | JN698581 |  |
| 18 | *L. paratatei* | P.86407 | JN698582 |  |
| 18 | *L. paratatei* | P.86408 | JN698583 | JQ624939 |
| 18 | *L. paratatei* | P.86409 | JN698584 | JQ624940 |
| 41 | *L. paratatei* | P.86392 | JN698567 |  |
| 41 | *L. paratatei* | P.86393 | JN698568 |  |
| 41 | *L. paratatei* | P.86394 | JN698569 |  |
| 41 | *L. paratatei* | P.86395 | JN698570 |  |
| 41 | *L. paratatei* | P.86396 | JN698571 |  |
| 41 | *L. paratatei* | P.86410 | JN698585 | JQ624941 |
| 41 | *L. paratatei* | P.86411 | JN698586 | JQ624942 |
| 41 | *L. paratatei* | P.86412 | JN698587 |  |
| 41 | *L. paratatei* | P.86413 | JN698588 |  |
| 41 | *L. paratatei* | P.86414 | JN698589 | JQ624943 |
| 41 | *L. paratatei* | P.86417 | JN698592 |  |
| 41 | *L. paratatei* | P.86418 | JN698593 |  |
| 42 | *L. paratatei* | P.86397 | JN698572 |  |
| 42 | *L. paratatei* | P.86398 | JN698573 |  |
| 42 | *L. paratatei* | P.86399 | JN698574 |  |
| 42 | *L. paratatei* | P.86419 | JN698594 |  |
| 42 | *L. paratatei* | P.86420 | JN698595 |  |
| 43 | *L. paratatei* | P.86400 | JN698575 |  |
| 43 | *L. paratatei* | P.86401 | JN698576 |  |
| 43 | *L. paratatei* | P.86415 | JN698590 |  |
| 43 | *L. paratatei* | P.86416 | JN698591 |  |
| 54 | *L. paratatei* | P.84210 | HQ717788 |  |
| 54 | *L. paratatei* | P.84211 | HQ717789 |  |
| 54 | *L. paratatei* | P.86386 | JN698561 |  |
| 54 | *L. paratatei* | P.86387 | JN698562 |  |
| 54 | *L. paratatei* | P.86388 | JN698563 |  |
| 39a | *L. paratatei* | P.84204 | HQ717782 |  |
| 39a | *L. paratatei* | P.84205 | HQ717783 |  |
| 39c | *L. paratatei* | P.84206 | HQ717784 |  |
| 39c | *L. paratatei* | P.84207 | HQ717785 | JQ624936 |
| 39c | *L. paratatei* | P.84208 | HQ717786 |  |
| 39c | *L. paratatei* | P.84209 | HQ717787 |  |
| 39c | *L. paratatei* | P.86389 | JN698564 |  |
| 39c | *L. paratatei* | P.86390 | JN698565 |  |
| 39c | *L. paratatei* | P.86391 | JN698566 | JQ624935 |
| 3 | *L. parvispinus* | P.85014 | JF966715 |  |
| 3 | *L. parvispinus* | P.85015 | JN698273 |  |
| 3 | *L. parvispinus* | P.85016 | JN698274 | JQ624886 |
| 3 | *L. parvispinus* | P.85017 | JN698275 |  |
| 4 | *L. parvispinus* | P.84191 | HQ717772 |  |
| 4 | *L. parvispinus* | P.84995 | JN698254 | JQ624883 |
| 4 | *L. parvispinus* | P.84996 | JN698255 | JQ624885 |
| 4 | *L. parvispinus* | P.84997 | JN698256 |  |
| 4 | *L. parvispinus* | Lim122* | JN698246 |  |
| 4 | *L. parvispinus* | Lim123* | JN698247 |  |
| 4 | *L. parvispinus* | Lim124* | JN698248 |  |
| 4 | *L. parvispinus* | Lim129* | JN698251 |  |
| 4 | *L. parvispinus* | Lim131* | JN698252 |  |
| 4 | *L. parvispinus* | Lim132* | JN698253 |  |
| 5 | *L. parvispinus* | P.85021 | JN698279 |  |
| 6 | *L. parvispinus* | P.84192 | HQ717773 | JQ624887 |
| 6 | *L. parvispinus* | P.85018 | JN698276 |  |
| 8 | *L. parvispinus* | P.85019 | JN698277 |  |
| 8 | *L. parvispinus* | P.85020 | JN698278 |  |
| 9 | *L. parvispinus* | P.85027 | JN698283 |  |
| 9 | *L. parvispinus* | P.85028 | JN698284 |  |
| 9 | *L. parvispinus* | P.85029 | JF966718 |  |
| 9 | *L. parvispinus* | P.85030 | JN698285 | JQ624882 |
| 9 | *L. parvispinus* | P.85031 | JN698286 |  |
| 10 | *L. parvispinus* | P.84185 | HQ717766 |  |
| 10 | *L. parvispinus* | P.85032 | JN698287 |  |
| 10 | *L. parvispinus* | P.85033 | JN698288 |  |
| 10 | *L. parvispinus* | P.85077 | JN698332 |  |
| 10 | *L. parvispinus* | P.85078 | JN698333 |  |
| 10 | *L. parvispinus* | P.86357 | JN698370 |  |
| 21 | *L. parvispinus* | P.85022 | JF966716 | JQ624889 |
| 21 | *L. parvispinus* | P.85023 | JF966717 |  |
| 21 | *L. parvispinus* | P.85024 | JN698280 |  |
| 21 | *L. parvispinus* | P.85025 | JN698281 | JQ624890 |
| 21 | *L. parvispinus* | P.85026 | JN698282 |  |
| 22 | *L. parvispinus* | Egg44 | JQ624888 | HQ717744 |
| 22 | *L. parvispinus* | Egg46 | HQ717745 |  |
| 23 | *L. parvispinus* | P.84183 | HQ717764 |  |
| 24 | *L. parvispinus* | P.84184 | HQ717765 |  |
| 30 | *L. parvispinus* | Egg15 | JN698243 |  |
| 32 | *L. parvispinus* | P.85050 | JN698305 |  |
| 32 | *L. parvispinus* | P.85051 | JN698306 |  |
| 32 | *L. parvispinus* | P.85052 | JN698307 |  |
| 32 | *L. parvispinus* | P.85053 | JN698308 |  |
| 32 | *L. parvispinus* | P.85054 | JN698309 |  |
| 33 | *L. parvispinus* | P.84182 | HQ717763 |  |
| 33 | *L. parvispinus* | P.85091 | JN698346 |  |
| 36 | *L. parvispinus* | P.84180 | FJ830344 | JQ624893 |
| 36 | *L. parvispinus* | P.85100 | JN698355 |  |
| 36 | *L. parvispinus* | P.85101 | JN698356 |  |
| 40 | *L. parvispinus* | P.85065 | JN698320 |  |
| 40 | *L. parvispinus* | P.85066 | JN698321 |  |
| 40 | *L. parvispinus* | P.85067 | JN698322 |  |
| 40 | *L. parvispinus* | P.85068 | JN698323 |  |
| 40 | *L. parvispinus* | P.85069 | JN698324 |  |
| 43 | *L. parvispinus* | P.85079 | JN698334 |  |
| 43 | *L. parvispinus* | P.85080 | JN698335 |  |
| 43 | *L. parvispinus* | P.85081 | JN698336 |  |
| 43 | *L. parvispinus* | P.85082 | JN698337 |  |
| 43 | *L. parvispinus* | P.85083 | JN698338 |  |
| 43 | *L. parvispinus* | P.86347 | JN698360 |  |
| 43 | *L. parvispinus* | P.86348 | JN698361 |  |
| 43 | *L. parvispinus* | P.86349 | JN698362 |  |
| 43 | *L. parvispinus* | P.86350 | JN698363 |  |
| 43 | *L. parvispinus* | P.86351 | JN698364 |  |
| 43 | *L. parvispinus* | P.86352 | JN698365 |  |
| 43 | *L. parvispinus* | P.86353 | JN698366 |  |
| 43 | *L. parvispinus* | P.86354 | JN698367 |  |
| 43 | *L. parvispinus* | P.86355 | JN698368 | JQ624897 |
| 43 | *L. parvispinus* | P.86356 | JN698369 |  |
| 45 | *L. parvispinus* | P.85045 | JN698300 |  |
| 45 | *L. parvispinus* | P.85046 | JN698301 |  |
| 45 | *L. parvispinus* | P.85047 | JN698302 |  |
| 45 | *L. parvispinus* | P.85048 | JN698303 |  |
| 45 | *L. parvispinus* | P.85049 | JN698304 |  |
| 46 | *L. parvispinus* | P.85084 | JN698339 |  |
| 46 | *L. parvispinus* | P.85085 | JN698340 | JQ624892 |
| 46 | *L. parvispinus* | P.85086 | JN698341 |  |
| 46 | *L. parvispinus* | P.85087 | JN698342 |  |
| 46 | *L. parvispinus* | P.85088 | JN698343 |  |
| 46 | *L. parvispinus* | P.86362 | JN698375 |  |
| 46 | *L. parvispinus* | P.86363 | JN698376 |  |
| 47 | *L. parvispinus* | P.85008 | JN698267 |  |
| 47 | *L. parvispinus* | P.85009 | JN698268 |  |
| 47 | *L. parvispinus* | P.85010 | JN698269 |  |
| 47 | *L. parvispinus* | P.85011 | JN698270 |  |
| 47 | *L. parvispinus* | P.85012 | JN698271 |  |
| 48 | *L. parvispinus* | P.85005 | JN698264 |  |
| 48 | *L. parvispinus* | P.85006 | JN698265 |  |
| 48 | *L. parvispinus* | P.85007 | JN698266 |  |
| 49 | *L. parvispinus* | P.80896 | HQ717751 |  |
| 49 | *L. parvispinus* | P.80897 | HQ717752 |  |
| 49 | *L. parvispinus* | P.80898 | HQ717753 |  |
| 49 | *L. parvispinus* | P.85089 | JN698344 |  |
| 49 | *L. parvispinus* | P.85090 | JN698345 |  |
| 49 | *L. parvispinus* | P.86364 | JN698377 |  |
| 49 | *L. parvispinus* | P.86365 | JN698378 |  |
| 49 | *L. parvispinus* | P.86366 | JN698379 |  |
| 49 | *L. parvispinus* | P.86367 | JN698380 |  |
| 53 | *L. parvispinus* | P.85013 | JN698272 |  |
| 54 | *L. parvispinus* | P.85060 | JN698315 |  |
| 54 | *L. parvispinus* | P.85061 | JN698316 |  |
| 54 | *L. parvispinus* | P.85062 | JN698317 |  |
| 54 | *L. parvispinus* | P.85063 | JN698318 |  |
| 54 | *L. parvispinus* | P.85064 | JN698319 |  |
| 56 | *L. parvispinus* | P.85042 | JN698297 |  |
| 56 | *L. parvispinus* | P.85043 | JN698298 |  |
| 56 | *L. parvispinus* | P.85044 | JN698299 |  |
| 62 | *L. parvispinus* | P.85055 | JN698310 |  |
| 62 | *L. parvispinus* | P.85056 | JN698311 |  |
| 62 | *L. parvispinus* | P.85057 | JN698312 | JQ624891 |
| 62 | *L. parvispinus* | P.85058 | JN698313 |  |
| 62 | *L. parvispinus* | P.85059 | JN698314 |  |
| 62 | *L. parvispinus* | P.86358 | JN698371 |  |
| 62 | *L. parvispinus* | P.86359 | JN698372 |  |
| 62 | *L. parvispinus* | P.86360 | JN698373 |  |
| 62 | *L. parvispinus* | P.86361 | JN698374 |  |
| 69 | *L. parvispinus* | P.85092 | JN698347 |  |
| 69 | *L. parvispinus* | P.85093 | JN698348 |  |
| 69 | *L. parvispinus* | P.85094 | JN698349 | JQ624894 |
| 69 | *L. parvispinus* | P.85095 | JN698350 |  |
| 69 | *L. parvispinus* | P.86368 | JN698381 |  |
| 69 | *L. parvispinus* | P.86369 | JN698382 | JQ624898 |
| 69 | *L. parvispinus* | P.86370 | JN698383 |  |
| 72 | *L. parvispinus* | P.85096 | JN698351 | JQ624895 |
| 72 | *L. parvispinus* | P.85097 | JN698352 | JQ624896 |
| 72 | *L. parvispinus* | P.85098 | JN698353 |  |
| 72 | *L. parvispinus* | P.85099 | JN698354 |  |
| 37a | *L. parvispinus* | P.85034 | JN698289 |  |
| 37a | *L. parvispinus* | P.85035 | JN698290 |  |
| 37a | *L. parvispinus* | P.85036 | JN698291 |  |
| 37a | *L. parvispinus* | P.85037 | JN698292 |  |
| 37a | *L. parvispinus* | P.85038 | JN698293 |  |
| 37b | *L. parvispinus* | P.86371 | JN698384 |  |
| 39b | *L. parvispinus* | P.86344 | JN698357 |  |
| 39b | *L. parvispinus* | P.86345 | JN698358 |  |
| 39b | *L. parvispinus* | P.86346 | JN698359 |  |
| 39b | *L. parvispinus* | Egg06 | JN698239 |  |
| 39b | *L. parvispinus* | Egg07 | JN698240 |  |
| 39b | *L. parvispinus* | Egg08 | JN698241 |  |
| 39b | *L. parvispinus* | Egg09 | JN698242 |  |
| 39b | *L. parvispinus* | Egg10 | HQ717743 |  |
| 39b | *L. parvispinus* | Egg18 | JN698244 |  |
| 39b | *L. parvispinus* | Egg19 | JN698245 |  |
| 39c | *L. parvispinus* | P.85039 | JN698294 |  |
| 39c | *L. parvispinus* | P.85040 | JN698295 |  |
| 39c | *L. parvispinus* | P.85041 | JN698296 |  |
| 39c | *L. parvispinus* | P.85070 | JN698325 |  |
| 39c | *L. parvispinus* | P.85071 | JN698326 |  |
| 39c | *L. parvispinus* | P.85072 | JN698327 |  |
| 39c | *L. parvispinus* | P.85073 | JN698328 |  |
| 39c | *L. parvispinus* | P.85074 | JN698329 |  |
| 39c | *L. parvispinus* | P.85075 | JN698330 |  |
| 39c | *L. parvispinus* | P.85076 | JN698331 |  |
| 65a | *L. parvispinus* | P.84998 | JN698257 |  |
| 65a | *L. parvispinus* | P.84999 | JN698258 |  |
| 65a | *L. parvispinus* | P.85000 | JN698259 |  |
| 65a | *L. parvispinus* | P.85001 | JN698260 |  |
| 7a | *L. parvispinus* | P.84189 | HQ717770 |  |
| 7a | *L. parvispinus* | P.84190 | HQ717771 |  |
| 7a | *L. parvispinus* | P.85002 | JN698261 |  |
| 7a | *L. parvispinus* | P.85003 | JN698262 |  |
| 7a | *L. parvispinus* | P.85004 | JN698263 |  |
| 7b | *L. parvispinus* | P.84186 | HQ717767 | JQ624881 |
| 7b | *L. parvispinus* | P.84187 | HQ717768 | JQ624884 |
| 7b | *L. parvispinus* | P.84188 | HQ717769 |  |
| 7b | *L. parvispinus* | Lim125* | JN698249 |  |
| 7b | *L. parvispinus* | Lim128* | JN698250 |  |
| 2 | *L. tatei* | P.87801 | JQ424942 | JQ624900 |
| 15 | *L. tatei* | P.85864 | JF966719 |  |
| 15 | *L. tatei* | P.85865 | JF966720 |  |
| 15 | *L. tatei* | P.85866 | JF966721 | JQ624910 |
| 15 | *L. tatei* | P.85867 | JF966722 |  |
| 15 | *L. tatei* | P.85868 | JF966723 |  |
| 15 | *L. tatei* | P.85869 | JF966724 |  |
| 15 | *L. tatei* | P.85870 | JF966725 |  |
| 15 | *L. tatei* | P.85871 | JF966726 |  |
| 15 | *L. tatei* | P.85872 | JF966727 |  |
| 15 | *L. tatei* | P.85873 | JF966728 |  |
| 20 | *L. tatei* | P.86378 | JN698553 | JQ624912 |
| 20 | *L. tatei* | P.86379 | JN698554 | JQ624913 |
| 20 | *L. tatei* | P.86380 | JN698555 |  |
| 20 | *L. tatei* | P.86381 | JN698556 |  |
| 20 | *L. tatei* | P.86382 | JN698557 |  |
| 23 | *L. tatei* | P.84197 | HQ717776 |  |
| 23 | *L. tatei* | P.84198 | FJ830345 | JQ624907 |
| 23 | *L. tatei* | P.85172 | JN698544 |  |
| 23 | *L. tatei* | P.85174 | JN698546 |  |
| 25 | *L. tatei* | P.84199 | HQ717777 |  |
| 25 | *L. tatei* | P.84200 | HQ717778 |  |
| 25 | *L. tatei* | P.84201 | HQ717779 | JQ624908 |
| 25 | *L. tatei* | P.84202 | HQ717780 |  |
| 25 | *L. tatei* | P.84203 | HQ717781 | JQ624909 |
| 25 | *L. tatei* | P.85173 | JN698545 |  |
| 28 | *L. tatei* | P.85168 | JN698540 | JQ624901 |
| 28 | *L. tatei* | P.85169 | JN698541 | JQ624902 |
| 28 | *L. tatei* | P.86375 | JN698550 | JQ624911 |
| 28 | *L. tatei* | P.86376 | JN698551 |  |
| 31 | *L. tatei* | P.85162 | JN698534 | JQ624903 |
| 31 | *L. tatei* | P.85163 | JN698535 | JQ624904 |
| 32 | *L. tatei* | P.85164 | JN698536 | JQ624905 |
| 32 | *L. tatei* | P.85165 | JN698537 | JQ624906 |
| 32 | *L. tatei* | P.86372 | JN698547 |  |
| 32 | *L. tatei* | P.86373 | JN698548 |  |
| 32 | *L. tatei* | P.86374 | JN698549 |  |
| 34 | *L. tatei* | P.85170 | JN698542 |  |
| 35 | *L. tatei* | P.85166 | JN698538 |  |
| 35 | *L. tatei* | P.85167 | JN698539 |  |
| 35 | *L. tatei* | P.86377 | JN698552 |  |
| 36 | *L. tatei* | P.84195 | HQ717774 |  |
| 36 | *L. tatei* | P.84196 | HQ717775 |  |
| 43 | *L. tatei* | P.86383 | JN698558 |  |
| 44 | *L. tatei* | P.85171 | JN698543 |  |
| 51 | *L. tatei* | P.86385 | JN698560 |  |
| 52 | *L. tatei* | P.86384 | JN698559 |  |
| 65a | *L. tatei* | P.85161 | JN698533 |  |

Supplement 2: Distribution of haplotypes of *L. parvispinus* for all localities. The sum of haplotypes for each locality equals the number of specimens studied form each locality. The haplotype names correspond to Fig. 2 and the locality names to Fig.1 and Table 1.

|  | | Locality | 3 | 4 | 5 | 6 | 7 | 8 | 9 | 10 | 21 | 22 | 23 | 24 | 30 | 32 | 33 | 36 | 37 | 39 | 40 | 43 | 45 | 46 | 47 | 48 | 49 | 53 | 54 | 56 | 62 | 65 | 69 | 72 |
| --- | --- | --- | --- | --- | --- | --- | --- | --- | --- | --- | --- | --- | --- | --- | --- | --- | --- | --- | --- | --- | --- | --- | --- | --- | --- | --- | --- | --- | --- | --- | --- | --- | --- | --- |
| Haplotype number | | Sum of haplotypes | 4 | 10 | 1 | 2 | 10 | 2 | 5 | 6 | 5 | 2 | 1 | 1 | 1 | 5 | 2 | 3 | 6 | 18 | 5 | 15 | 5 | 7 | 5 | 3 | 9 | 1 | 5 | 3 | 9 | 4 | 7 | 4 |
| Lp1 | | 3 | 0 | 0 | 0 | 0 | 0 | 0 | 0 | 0 | 0 | 0 | 0 | 0 | 0 | 0 | 0 | 0 | 0 | 1 | 0 | 0 | 0 | 0 | 0 | 0 | 2 | 0 | 0 | 0 | 0 | 0 | 0 | 0 |
| Lp2 | | 1 | 0 | 0 | 0 | 0 | 0 | 0 | 0 | 0 | 0 | 0 | 0 | 0 | 0 | 0 | 0 | 0 | 0 | 0 | 0 | 1 | 0 | 0 | 0 | 0 | 0 | 0 | 0 | 0 | 0 | 0 | 0 | 0 |
| Lp3 | | 2 | 0 | 0 | 0 | 0 | 0 | 0 | 0 | 0 | 0 | 0 | 0 | 0 | 0 | 1 | 0 | 0 | 0 | 0 | 0 | 0 | 0 | 1 | 0 | 0 | 0 | 0 | 0 | 0 | 0 | 0 | 0 | 0 |
| Lp4 | | 2 | 0 | 0 | 0 | 0 | 0 | 0 | 0 | 0 | 0 | 0 | 0 | 0 | 0 | 0 | 0 | 0 | 1 | 0 | 0 | 0 | 0 | 0 | 0 | 0 | 0 | 0 | 0 | 0 | 0 | 0 | 1 | 0 |
| Lp5 | | 1 | 0 | 0 | 0 | 0 | 0 | 0 | 0 | 0 | 0 | 0 | 0 | 0 | 0 | 0 | 0 | 0 | 0 | 0 | 0 | 0 | 0 | 0 | 0 | 0 | 0 | 0 | 0 | 0 | 1 | 0 | 0 | 0 |
| Lp6 | | 31 | 0 | 0 | 0 | 0 | 0 | 0 | 0 | 0 | 0 | 0 | 0 | 1 | 1 | 3 | 0 | 1 | 2 | 6 | 3 | 7 | 1 | 0 | 3 | 1 | 0 | 0 | 1 | 0 | 0 | 1 | 0 | 0 |
| Lp7 | | 3 | 0 | 0 | 0 | 0 | 0 | 0 | 0 | 0 | 0 | 0 | 0 | 0 | 0 | 0 | 0 | 0 | 0 | 0 | 0 | 0 | 3 | 0 | 0 | 0 | 0 | 0 | 0 | 0 | 0 | 0 | 0 | 0 |
| Lp8 | | 1 | 0 | 0 | 0 | 0 | 0 | 0 | 0 | 0 | 0 | 0 | 0 | 0 | 0 | 0 | 0 | 0 | 0 | 1 | 0 | 0 | 0 | 0 | 0 | 0 | 0 | 0 | 0 | 0 | 0 | 0 | 0 | 0 |
| Lp9 | | 1 | 0 | 0 | 0 | 0 | 0 | 0 | 0 | 0 | 0 | 0 | 0 | 0 | 0 | 0 | 0 | 0 | 0 | 0 | 0 | 0 | 0 | 1 | 0 | 0 | 0 | 0 | 0 | 0 | 0 | 0 | 0 | 0 |
| Lp10 | | 1 | 0 | 0 | 0 | 0 | 0 | 0 | 0 | 0 | 0 | 0 | 0 | 0 | 0 | 0 | 0 | 0 | 0 | 0 | 0 | 0 | 0 | 0 | 0 | 0 | 0 | 0 | 0 | 0 | 0 | 0 | 1 | 0 |
| Lp11 | | 1 | 0 | 0 | 0 | 0 | 0 | 0 | 0 | 0 | 0 | 0 | 0 | 0 | 0 | 0 | 0 | 0 | 0 | 0 | 0 | 1 | 0 | 0 | 0 | 0 | 0 | 0 | 0 | 0 | 0 | 0 | 0 | 0 |
|  | Lp12 | 1 | 0 | 0 | 0 | 0 | 0 | 0 | 0 | 0 | 0 | 0 | 0 | 0 | 0 | 0 | 0 | 0 | 0 | 0 | 0 | 0 | 0 | 0 | 0 | 0 | 1 | 0 | 0 | 0 | 0 | 0 | 0 | 0 |
|  | Lp13 | 1 | 0 | 0 | 0 | 0 | 0 | 0 | 0 | 0 | 0 | 0 | 0 | 0 | 0 | 0 | 0 | 0 | 0 | 0 | 0 | 1 | 0 | 0 | 0 | 0 | 0 | 0 | 0 | 0 | 0 | 0 | 0 | 0 |
|  | Lp14 | 2 | 0 | 0 | 0 | 0 | 0 | 0 | 0 | 0 | 0 | 0 | 0 | 0 | 0 | 0 | 0 | 0 | 0 | 1 | 0 | 0 | 0 | 0 | 0 | 0 | 0 | 0 | 0 | 0 | 0 | 1 | 0 | 0 |
|  | Lp15 | 1 | 0 | 0 | 0 | 0 | 0 | 0 | 0 | 0 | 0 | 0 | 0 | 0 | 0 | 0 | 0 | 0 | 0 | 0 | 0 | 1 | 0 | 0 | 0 | 0 | 0 | 0 | 0 | 0 | 0 | 0 | 0 | 0 |
|  | Lp16 | 1 | 0 | 0 | 0 | 0 | 0 | 0 | 0 | 0 | 0 | 0 | 0 | 0 | 0 | 0 | 0 | 0 | 0 | 0 | 0 | 0 | 0 | 0 | 0 | 0 | 0 | 0 | 0 | 0 | 0 | 0 | 1 | 0 |
|  | Lp17 | 2 | 0 | 0 | 0 | 0 | 0 | 0 | 0 | 0 | 0 | 0 | 0 | 0 | 0 | 0 | 0 | 0 | 0 | 1 | 0 | 0 | 0 | 0 | 0 | 0 | 0 | 0 | 0 | 1 | 0 | 0 | 0 | 0 |
|  | Lp18 | 2 | 0 | 0 | 0 | 0 | 0 | 0 | 0 | 0 | 0 | 0 | 0 | 0 | 0 | 0 | 0 | 0 | 0 | 1 | 0 | 0 | 0 | 0 | 0 | 0 | 1 | 0 | 0 | 0 | 0 | 0 | 0 | 0 |
|  | Lp19 | 2 | 0 | 0 | 0 | 0 | 0 | 0 | 0 | 0 | 0 | 0 | 0 | 0 | 0 | 0 | 0 | 0 | 0 | 0 | 0 | 0 | 0 | 0 | 0 | 1 | 0 | 0 | 0 | 0 | 0 | 0 | 1 | 0 |
|  | Lp20 | 13 | 0 | 0 | 0 | 0 | 0 | 0 | 0 | 0 | 2 | 0 | 0 | 0 | 0 | 0 | 1 | 0 | 2 | 3 | 1 | 1 | 0 | 0 | 1 | 1 | 0 | 0 | 1 | 0 | 0 | 0 | 0 | 0 |
|  | Lp21 | 3 | 0 | 0 | 0 | 0 | 0 | 0 | 0 | 0 | 0 | 0 | 0 | 0 | 0 | 0 | 0 | 0 | 1 | 0 | 0 | 0 | 0 | 1 | 0 | 0 | 1 | 0 | 0 | 0 | 0 | 0 | 0 | 0 |
|  | Lp22 | 1 | 0 | 0 | 0 | 0 | 0 | 0 | 0 | 0 | 0 | 1 | 0 | 0 | 0 | 0 | 0 | 0 | 0 | 0 | 0 | 0 | 0 | 0 | 0 | 0 | 0 | 0 | 0 | 0 | 0 | 0 | 0 | 0 |
|  | Lp23 | 1 | 0 | 0 | 0 | 0 | 0 | 0 | 0 | 0 | 0 | 0 | 0 | 0 | 0 | 0 | 0 | 0 | 0 | 1 | 0 | 0 | 0 | 0 | 0 | 0 | 0 | 0 | 0 | 0 | 0 | 0 | 0 | 0 |
|  | Lp24 | 6 | 0 | 0 | 0 | 0 | 0 | 0 | 0 | 0 | 0 | 0 | 0 | 0 | 0 | 0 | 0 | 0 | 0 | 1 | 1 | 0 | 0 | 1 | 0 | 0 | 2 | 0 | 1 | 0 | 0 | 0 | 0 | 0 |
|  | Lp25 | 1 | 0 | 0 | 0 | 0 | 0 | 0 | 0 | 0 | 0 | 0 | 0 | 0 | 0 | 0 | 0 | 0 | 0 | 0 | 0 | 0 | 0 | 1 | 0 | 0 | 0 | 0 | 0 | 0 | 0 | 0 | 0 | 0 |
|  | Lp26 | 1 | 0 | 0 | 0 | 0 | 0 | 0 | 0 | 0 | 0 | 0 | 0 | 0 | 0 | 1 | 0 | 0 | 0 | 0 | 0 | 0 | 0 | 0 | 0 | 0 | 0 | 0 | 0 | 0 | 0 | 0 | 0 | 0 |
|  | Lp27 | 1 | 0 | 0 | 0 | 0 | 0 | 0 | 0 | 0 | 0 | 0 | 0 | 0 | 0 | 0 | 0 | 0 | 0 | 0 | 0 | 0 | 0 | 0 | 0 | 0 | 0 | 0 | 0 | 0 | 0 | 0 | 1 | 0 |
|  | Lp28 | 1 | 0 | 0 | 0 | 0 | 0 | 0 | 0 | 0 | 0 | 0 | 0 | 0 | 0 | 0 | 0 | 0 | 0 | 0 | 0 | 0 | 0 | 0 | 0 | 0 | 0 | 0 | 0 | 0 | 0 | 1 | 0 | 0 |
|  | Lp29 | 2 | 0 | 0 | 0 | 0 | 0 | 0 | 0 | 0 | 0 | 0 | 0 | 0 | 0 | 0 | 0 | 2 | 0 | 0 | 0 | 0 | 0 | 0 | 0 | 0 | 0 | 0 | 0 | 0 | 0 | 0 | 0 | 0 |
|  | Lp30 | 1 | 0 | 0 | 0 | 0 | 0 | 0 | 0 | 0 | 0 | 0 | 0 | 0 | 0 | 0 | 0 | 0 | 0 | 0 | 0 | 0 | 0 | 0 | 0 | 0 | 0 | 0 | 1 | 0 | 0 | 0 | 0 | 0 |
|  | Lp31 | 1 | 0 | 0 | 0 | 0 | 0 | 0 | 0 | 0 | 0 | 0 | 0 | 0 | 0 | 0 | 0 | 0 | 0 | 0 | 0 | 0 | 0 | 0 | 1 | 0 | 0 | 0 | 0 | 0 | 0 | 0 | 0 | 0 |
|  | Lp32 | 1 | 0 | 0 | 0 | 0 | 0 | 0 | 0 | 0 | 0 | 0 | 0 | 0 | 0 | 0 | 0 | 0 | 0 | 0 | 0 | 0 | 0 | 0 | 0 | 0 | 0 | 0 | 0 | 0 | 1 | 0 | 0 | 0 |
|  | Lp33 | 1 | 0 | 0 | 0 | 0 | 0 | 0 | 0 | 0 | 0 | 0 | 0 | 0 | 0 | 0 | 0 | 0 | 0 | 0 | 0 | 1 | 0 | 0 | 0 | 0 | 0 | 0 | 0 | 0 | 0 | 0 | 0 | 0 |
|  | Lp34 | 1 | 0 | 0 | 0 | 0 | 0 | 0 | 0 | 0 | 0 | 1 | 0 | 0 | 0 | 0 | 0 | 0 | 0 | 0 | 0 | 0 | 0 | 0 | 0 | 0 | 0 | 0 | 0 | 0 | 0 | 0 | 0 | 0 |
|  | Lp35 | 1 | 0 | 0 | 0 | 0 | 0 | 0 | 0 | 0 | 0 | 0 | 0 | 0 | 0 | 0 | 0 | 0 | 0 | 0 | 0 | 0 | 0 | 0 | 0 | 0 | 0 | 0 | 0 | 1 | 0 | 0 | 0 | 0 |
|  | Lp36 | 1 | 0 | 0 | 0 | 0 | 0 | 0 | 0 | 0 | 0 | 0 | 0 | 0 | 0 | 0 | 0 | 0 | 0 | 0 | 0 | 0 | 1 | 0 | 0 | 0 | 0 | 0 | 0 | 0 | 0 | 0 | 0 | 0 |
|  | Lp37 | 1 | 0 | 0 | 0 | 0 | 0 | 0 | 0 | 0 | 0 | 0 | 0 | 0 | 0 | 0 | 1 | 0 | 0 | 0 | 0 | 0 | 0 | 0 | 0 | 0 | 0 | 0 | 0 | 0 | 0 | 0 | 0 | 0 |
|  | Lp38 | 4 | 0 | 0 | 0 | 0 | 0 | 0 | 0 | 0 | 0 | 0 | 0 | 0 | 0 | 0 | 0 | 0 | 0 | 0 | 0 | 0 | 0 | 0 | 0 | 0 | 0 | 0 | 0 | 0 | 4 | 0 | 0 | 0 |
|  | Lp39 | 1 | 0 | 0 | 0 | 0 | 0 | 0 | 0 | 0 | 0 | 0 | 0 | 0 | 0 | 0 | 0 | 0 | 0 | 0 | 0 | 0 | 0 | 0 | 0 | 0 | 0 | 0 | 0 | 0 | 1 | 0 | 0 | 0 |
|  | Lp40 | 1 | 0 | 0 | 0 | 0 | 0 | 0 | 0 | 0 | 0 | 0 | 0 | 0 | 0 | 0 | 0 | 0 | 0 | 0 | 0 | 0 | 0 | 0 | 0 | 0 | 1 | 0 | 0 | 0 | 0 | 0 | 0 | 0 |
|  | Lp41 | 1 | 0 | 0 | 0 | 0 | 0 | 0 | 0 | 0 | 0 | 0 | 0 | 0 | 0 | 0 | 0 | 0 | 0 | 0 | 0 | 0 | 0 | 0 | 0 | 0 | 0 | 0 | 1 | 0 | 0 | 0 | 0 | 0 |
|  | Lp42 | 1 | 0 | 0 | 0 | 0 | 0 | 0 | 0 | 0 | 0 | 0 | 0 | 0 | 0 | 0 | 0 | 0 | 0 | 0 | 0 | 0 | 0 | 1 | 0 | 0 | 0 | 0 | 0 | 0 | 0 | 0 | 0 | 0 |
|  | Lp43 | 1 | 0 | 0 | 0 | 0 | 0 | 0 | 0 | 0 | 0 | 0 | 0 | 0 | 0 | 0 | 0 | 0 | 0 | 0 | 0 | 0 | 0 | 0 | 0 | 0 | 0 | 0 | 0 | 0 | 0 | 0 | 1 | 0 |
|  | Lp44 | 1 | 0 | 0 | 0 | 0 | 0 | 0 | 0 | 0 | 0 | 0 | 0 | 0 | 0 | 0 | 0 | 0 | 0 | 1 | 0 | 0 | 0 | 0 | 0 | 0 | 0 | 0 | 0 | 0 | 0 | 0 | 0 | 0 |
|  | Lp45 | 1 | 0 | 0 | 0 | 0 | 0 | 0 | 0 | 0 | 0 | 0 | 0 | 0 | 0 | 0 | 0 | 0 | 0 | 0 | 0 | 0 | 0 | 0 | 0 | 0 | 0 | 0 | 0 | 0 | 0 | 1 | 0 | 0 |
|  | Lp46 | 6 | 0 | 0 | 0 | 0 | 0 | 0 | 0 | 0 | 3 | 0 | 1 | 0 | 0 | 0 | 0 | 0 | 0 | 0 | 0 | 2 | 0 | 0 | 0 | 0 | 0 | 0 | 0 | 0 | 0 | 0 | 0 | 0 |
|  | Lp47 | 1 | 0 | 0 | 0 | 0 | 0 | 0 | 0 | 0 | 0 | 0 | 0 | 0 | 0 | 0 | 0 | 0 | 0 | 0 | 0 | 0 | 0 | 0 | 0 | 0 | 0 | 1 | 0 | 0 | 0 | 0 | 0 | 0 |
|  | Lp48 | 2 | 0 | 0 | 0 | 0 | 0 | 0 | 0 | 0 | 0 | 0 | 0 | 0 | 0 | 0 | 0 | 0 | 0 | 0 | 0 | 0 | 0 | 0 | 0 | 0 | 0 | 0 | 0 | 0 | 1 | 0 | 1 | 0 |
|  | Lp49 | 1 | 0 | 0 | 0 | 0 | 0 | 0 | 0 | 0 | 0 | 0 | 0 | 0 | 0 | 0 | 0 | 0 | 0 | 1 | 0 | 0 | 0 | 0 | 0 | 0 | 0 | 0 | 0 | 0 | 0 | 0 | 0 | 0 |
|  | Lp50 | 1 | 0 | 0 | 0 | 0 | 0 | 0 | 0 | 0 | 0 | 0 | 0 | 0 | 0 | 0 | 0 | 0 | 0 | 0 | 0 | 0 | 0 | 0 | 0 | 0 | 0 | 0 | 0 | 0 | 1 | 0 | 0 | 0 |
|  | Lp51 | 1 | 0 | 0 | 0 | 0 | 0 | 0 | 0 | 0 | 0 | 0 | 0 | 0 | 0 | 0 | 0 | 0 | 0 | 0 | 0 | 0 | 0 | 0 | 0 | 0 | 0 | 0 | 0 | 0 | 0 | 0 | 0 | 1 |
|  | Lp52 | 1 | 0 | 0 | 0 | 0 | 0 | 0 | 0 | 0 | 0 | 0 | 0 | 0 | 0 | 0 | 0 | 0 | 0 | 0 | 0 | 0 | 0 | 1 | 0 | 0 | 0 | 0 | 0 | 0 | 0 | 0 | 0 | 0 |
|  | Lp53 | 2 | 0 | 0 | 0 | 0 | 0 | 0 | 0 | 0 | 0 | 0 | 0 | 0 | 0 | 0 | 0 | 0 | 0 | 0 | 0 | 0 | 0 | 0 | 0 | 0 | 1 | 0 | 0 | 1 | 0 | 0 | 0 | 0 |
|  | Lp54 | 2 | 0 | 0 | 0 | 0 | 0 | 0 | 0 | 0 | 0 | 0 | 0 | 0 | 0 | 0 | 0 | 0 | 0 | 0 | 0 | 0 | 0 | 0 | 0 | 0 | 0 | 0 | 0 | 0 | 0 | 0 | 0 | 2 |
|  | Lp55 | 1 | 0 | 0 | 0 | 0 | 0 | 0 | 0 | 0 | 0 | 0 | 0 | 0 | 0 | 0 | 0 | 0 | 0 | 0 | 0 | 0 | 0 | 0 | 0 | 0 | 0 | 0 | 0 | 0 | 0 | 0 | 0 | 1 |
|  | Lp56 | 4 | 0 | 0 | 0 | 0 | 4 | 0 | 0 | 0 | 0 | 0 | 0 | 0 | 0 | 0 | 0 | 0 | 0 | 0 | 0 | 0 | 0 | 0 | 0 | 0 | 0 | 0 | 0 | 0 | 0 | 0 | 0 | 0 |
|  | Lp57 | 4 | 0 | 1 | 0 | 0 | 3 | 0 | 0 | 0 | 0 | 0 | 0 | 0 | 0 | 0 | 0 | 0 | 0 | 0 | 0 | 0 | 0 | 0 | 0 | 0 | 0 | 0 | 0 | 0 | 0 | 0 | 0 | 0 |
|  | Lp58 | 1 | 0 | 0 | 1 | 0 | 0 | 0 | 0 | 0 | 0 | 0 | 0 | 0 | 0 | 0 | 0 | 0 | 0 | 0 | 0 | 0 | 0 | 0 | 0 | 0 | 0 | 0 | 0 | 0 | 0 | 0 | 0 | 0 |
|  | Lp59 | 3 | 0 | 3 | 0 | 0 | 0 | 0 | 0 | 0 | 0 | 0 | 0 | 0 | 0 | 0 | 0 | 0 | 0 | 0 | 0 | 0 | 0 | 0 | 0 | 0 | 0 | 0 | 0 | 0 | 0 | 0 | 0 | 0 |
|  | Lp60 | 1 | 0 | 0 | 0 | 0 | 0 | 0 | 1 | 0 | 0 | 0 | 0 | 0 | 0 | 0 | 0 | 0 | 0 | 0 | 0 | 0 | 0 | 0 | 0 | 0 | 0 | 0 | 0 | 0 | 0 | 0 | 0 | 0 |
|  | Lp61 | 2 | 0 | 2 | 0 | 0 | 0 | 0 | 0 | 0 | 0 | 0 | 0 | 0 | 0 | 0 | 0 | 0 | 0 | 0 | 0 | 0 | 0 | 0 | 0 | 0 | 0 | 0 | 0 | 0 | 0 | 0 | 0 | 0 |
|  | Lp62 | 2 | 0 | 0 | 0 | 0 | 2 | 0 | 0 | 0 | 0 | 0 | 0 | 0 | 0 | 0 | 0 | 0 | 0 | 0 | 0 | 0 | 0 | 0 | 0 | 0 | 0 | 0 | 0 | 0 | 0 | 0 | 0 | 0 |
|  | Lp63 | 1 | 0 | 0 | 0 | 1 | 0 | 0 | 0 | 0 | 0 | 0 | 0 | 0 | 0 | 0 | 0 | 0 | 0 | 0 | 0 | 0 | 0 | 0 | 0 | 0 | 0 | 0 | 0 | 0 | 0 | 0 | 0 | 0 |
|  | Lp64 | 2 | 2 | 0 | 0 | 0 | 0 | 0 | 0 | 0 | 0 | 0 | 0 | 0 | 0 | 0 | 0 | 0 | 0 | 0 | 0 | 0 | 0 | 0 | 0 | 0 | 0 | 0 | 0 | 0 | 0 | 0 | 0 | 0 |
|  | Lp65 | 1 | 0 | 1 | 0 | 0 | 0 | 0 | 0 | 0 | 0 | 0 | 0 | 0 | 0 | 0 | 0 | 0 | 0 | 0 | 0 | 0 | 0 | 0 | 0 | 0 | 0 | 0 | 0 | 0 | 0 | 0 | 0 | 0 |
|  | Lp66 | 8 | 0 | 0 | 0 | 1 | 1 | 2 | 4 | 0 | 0 | 0 | 0 | 0 | 0 | 0 | 0 | 0 | 0 | 0 | 0 | 0 | 0 | 0 | 0 | 0 | 0 | 0 | 0 | 0 | 0 | 0 | 0 | 0 |
|  | Lp67 | 2 | 2 | 0 | 0 | 0 | 0 | 0 | 0 | 0 | 0 | 0 | 0 | 0 | 0 | 0 | 0 | 0 | 0 | 0 | 0 | 0 | 0 | 0 | 0 | 0 | 0 | 0 | 0 | 0 | 0 | 0 | 0 | 0 |
|  | Lp68 | 1 | 0 | 1 | 0 | 0 | 0 | 0 | 0 | 0 | 0 | 0 | 0 | 0 | 0 | 0 | 0 | 0 | 0 | 0 | 0 | 0 | 0 | 0 | 0 | 0 | 0 | 0 | 0 | 0 | 0 | 0 | 0 | 0 |
|  | Lp69 | 1 | 0 | 1 | 0 | 0 | 0 | 0 | 0 | 0 | 0 | 0 | 0 | 0 | 0 | 0 | 0 | 0 | 0 | 0 | 0 | 0 | 0 | 0 | 0 | 0 | 0 | 0 | 0 | 0 | 0 | 0 | 0 | 0 |
|  | Lp70 | 5 | 0 | 1 | 0 | 0 | 0 | 0 | 0 | 4 | 0 | 0 | 0 | 0 | 0 | 0 | 0 | 0 | 0 | 0 | 0 | 0 | 0 | 0 | 0 | 0 | 0 | 0 | 0 | 0 | 0 | 0 | 0 | 0 |
|  | Lp71 | 1 | 0 | 0 | 0 | 0 | 0 | 0 | 0 | 1 | 0 | 0 | 0 | 0 | 0 | 0 | 0 | 0 | 0 | 0 | 0 | 0 | 0 | 0 | 0 | 0 | 0 | 0 | 0 | 0 | 0 | 0 | 0 | 0 |
|  | Lp72 | 1 | 0 | 0 | 0 | 0 | 0 | 0 | 0 | 1 | 0 | 0 | 0 | 0 | 0 | 0 | 0 | 0 | 0 | 0 | 0 | 0 | 0 | 0 | 0 | 0 | 0 | 0 | 0 | 0 | 0 | 0 | 0 | 0 |

Supplement Table 3: Distribution of haplotypes of *L. birchii* for all localities. The sum of haplotypes for each locality equals the number of specimens studied form each locality. The haplotype names correspond to Fig. 2 and the locality names to Fig.1 and Table 1.

|  | Locality | 1 | 7 | 10 | 11 | 12 | 13 | 14 | 16 | 17 | 18 | 19 | 20 | 24 | 26 | 27 | 28 | 29 | 31 | 32 | 33 | 34 | 37 | 38 | 45 | 46 | 48 | 50 | 51 | 52 | 53 | 54 | 55 | 56 | 57 | 58 | 59 | 60 | 61 | 62 | 63 | 64 | 65 | 66 | 67 | 68 | 69 | 70 | 71 |
| --- | --- | --- | --- | --- | --- | --- | --- | --- | --- | --- | --- | --- | --- | --- | --- | --- | --- | --- | --- | --- | --- | --- | --- | --- | --- | --- | --- | --- | --- | --- | --- | --- | --- | --- | --- | --- | --- | --- | --- | --- | --- | --- | --- | --- | --- | --- | --- | --- | --- |
| Haplotype number | Sum of haplotypes | 3 | 5 | 5 | 5 | 1 | 5 | 8 | 3 | 5 | 5 | 5 | 5 | 4 | 1 | 3 | 1 | 2 | 2 | 1 | 5 | 2 | 2 | 2 | 1 | 2 | 1 | 1 | 2 | 2 | 2 | 5 | 2 | 5 | 5 | 2 | 4 | 2 | 5 | 1 | 2 | 5 | 5 | 2 | 5 | 5 | 5 | 1 | 2 |
| Lb1 | 4 | 0 | 4 | 0 | 0 | 0 | 0 | 0 | 0 | 0 | 0 | 0 | 0 | 0 | 0 | 0 | 0 | 0 | 0 | 0 | 0 | 0 | 0 | 0 | 0 | 0 | 0 | 0 | 0 | 0 | 0 | 0 | 0 | 0 | 0 | 0 | 0 | 0 | 0 | 0 | 0 | 0 | 0 | 0 | 0 | 0 | 0 | 0 | 0 |
| Lb2 | 1 | 0 | 0 | 0 | 1 | 0 | 0 | 0 | 0 | 0 | 0 | 0 | 0 | 0 | 0 | 0 | 0 | 0 | 0 | 0 | 0 | 0 | 0 | 0 | 0 | 0 | 0 | 0 | 0 | 0 | 0 | 0 | 0 | 0 | 0 | 0 | 0 | 0 | 0 | 0 | 0 | 0 | 0 | 0 | 0 | 0 | 0 | 0 | 0 |
| Lb3 | 16 | 0 | 1 | 5 | 3 | 1 | 5 | 0 | 0 | 0 | 0 | 0 | 0 | 0 | 0 | 0 | 0 | 0 | 0 | 0 | 1 | 0 | 0 | 0 | 0 | 0 | 0 | 0 | 0 | 0 | 0 | 0 | 0 | 0 | 0 | 0 | 0 | 0 | 0 | 0 | 0 | 0 | 0 | 0 | 0 | 0 | 0 | 0 | 0 |
| Lb4 | 1 | 0 | 0 | 0 | 1 | 0 | 0 | 0 | 0 | 0 | 0 | 0 | 0 | 0 | 0 | 0 | 0 | 0 | 0 | 0 | 0 | 0 | 0 | 0 | 0 | 0 | 0 | 0 | 0 | 0 | 0 | 0 | 0 | 0 | 0 | 0 | 0 | 0 | 0 | 0 | 0 | 0 | 0 | 0 | 0 | 0 | 0 | 0 | 0 |
| Lb5 | 3 | 3 | 0 | 0 | 0 | 0 | 0 | 0 | 0 | 0 | 0 | 0 | 0 | 0 | 0 | 0 | 0 | 0 | 0 | 0 | 0 | 0 | 0 | 0 | 0 | 0 | 0 | 0 | 0 | 0 | 0 | 0 | 0 | 0 | 0 | 0 | 0 | 0 | 0 | 0 | 0 | 0 | 0 | 0 | 0 | 0 | 0 | 0 | 0 |
| Lb6 | 2 | 0 | 0 | 0 | 0 | 0 | 0 | 0 | 0 | 0 | 0 | 0 | 0 | 0 | 0 | 0 | 0 | 0 | 0 | 0 | 0 | 0 | 2 | 0 | 0 | 0 | 0 | 0 | 0 | 0 | 0 | 0 | 0 | 0 | 0 | 0 | 0 | 0 | 0 | 0 | 0 | 0 | 0 | 0 | 0 | 0 | 0 | 0 | 0 |
| Lb7 | 1 | 0 | 0 | 0 | 0 | 0 | 0 | 0 | 0 | 0 | 0 | 0 | 0 | 0 | 0 | 0 | 0 | 0 | 0 | 0 | 0 | 0 | 0 | 0 | 0 | 0 | 0 | 0 | 0 | 0 | 0 | 0 | 1 | 0 | 0 | 0 | 0 | 0 | 0 | 0 | 0 | 0 | 0 | 0 | 0 | 0 | 0 | 0 | 0 |
| Lb8 | 17 | 0 | 0 | 0 | 0 | 0 | 0 | 0 | 0 | 0 | 0 | 0 | 0 | 1 | 0 | 0 | 0 | 0 | 0 | 0 | 1 | 1 | 0 | 0 | 1 | 0 | 0 | 0 | 0 | 0 | 0 | 1 | 1 | 1 | 1 | 0 | 0 | 0 | 3 | 0 | 2 | 2 | 1 | 0 | 1 | 0 | 0 | 0 | 0 |
| Lb9 | 1 | 0 | 0 | 0 | 0 | 0 | 0 | 0 | 0 | 0 | 0 | 0 | 0 | 1 | 0 | 0 | 0 | 0 | 0 | 0 | 0 | 0 | 0 | 0 | 0 | 0 | 0 | 0 | 0 | 0 | 0 | 0 | 0 | 0 | 0 | 0 | 0 | 0 | 0 | 0 | 0 | 0 | 0 | 0 | 0 | 0 | 0 | 0 | 0 |
| Lb10 | 1 | 0 | 0 | 0 | 0 | 0 | 0 | 0 | 0 | 0 | 0 | 0 | 0 | 0 | 0 | 0 | 0 | 0 | 0 | 0 | 0 | 0 | 0 | 0 | 0 | 0 | 0 | 0 | 0 | 0 | 0 | 0 | 0 | 0 | 0 | 0 | 1 | 0 | 0 | 0 | 0 | 0 | 0 | 0 | 0 | 0 | 0 | 0 | 0 |
| Lb11 | 1 | 0 | 0 | 0 | 0 | 0 | 0 | 0 | 0 | 0 | 0 | 0 | 0 | 0 | 0 | 0 | 0 | 1 | 0 | 0 | 0 | 0 | 0 | 0 | 0 | 0 | 0 | 0 | 0 | 0 | 0 | 0 | 0 | 0 | 0 | 0 | 0 | 0 | 0 | 0 | 0 | 0 | 0 | 0 | 0 | 0 | 0 | 0 | 0 |
| Lb12 | 5 | 0 | 0 | 0 | 0 | 0 | 0 | 0 | 0 | 0 | 0 | 0 | 0 | 0 | 0 | 0 | 0 | 0 | 0 | 0 | 0 | 0 | 0 | 1 | 0 | 1 | 0 | 0 | 0 | 0 | 0 | 0 | 0 | 0 | 0 | 0 | 0 | 1 | 0 | 0 | 0 | 0 | 1 | 1 | 0 | 0 | 0 | 0 | 0 |
| Lb13 | 1 | 0 | 0 | 0 | 0 | 0 | 0 | 0 | 0 | 0 | 0 | 0 | 0 | 0 | 0 | 0 | 0 | 0 | 0 | 0 | 0 | 1 | 0 | 0 | 0 | 0 | 0 | 0 | 0 | 0 | 0 | 0 | 0 | 0 | 0 | 0 | 0 | 0 | 0 | 0 | 0 | 0 | 0 | 0 | 0 | 0 | 0 | 0 | 0 |
| Lb14 | 2 | 0 | 0 | 0 | 0 | 0 | 0 | 0 | 0 | 0 | 0 | 0 | 0 | 0 | 0 | 0 | 0 | 0 | 0 | 0 | 0 | 0 | 0 | 0 | 0 | 0 | 0 | 0 | 0 | 2 | 0 | 0 | 0 | 0 | 0 | 0 | 0 | 0 | 0 | 0 | 0 | 0 | 0 | 0 | 0 | 0 | 0 | 0 | 0 |
| Lb15 | 1 | 0 | 0 | 0 | 0 | 0 | 0 | 0 | 0 | 0 | 0 | 0 | 0 | 0 | 0 | 1 | 0 | 0 | 0 | 0 | 0 | 0 | 0 | 0 | 0 | 0 | 0 | 0 | 0 | 0 | 0 | 0 | 0 | 0 | 0 | 0 | 0 | 0 | 0 | 0 | 0 | 0 | 0 | 0 | 0 | 0 | 0 | 0 | 0 |
| Lb16 | 1 | 0 | 0 | 0 | 0 | 0 | 0 | 0 | 0 | 0 | 0 | 0 | 0 | 0 | 0 | 0 | 0 | 0 | 0 | 0 | 1 | 0 | 0 | 0 | 0 | 0 | 0 | 0 | 0 | 0 | 0 | 0 | 0 | 0 | 0 | 0 | 0 | 0 | 0 | 0 | 0 | 0 | 0 | 0 | 0 | 0 | 0 | 0 | 0 |
| Lb17 | 6 | 0 | 0 | 0 | 0 | 0 | 0 | 0 | 0 | 5 | 0 | 0 | 1 | 0 | 0 | 0 | 0 | 0 | 0 | 0 | 0 | 0 | 0 | 0 | 0 | 0 | 0 | 0 | 0 | 0 | 0 | 0 | 0 | 0 | 0 | 0 | 0 | 0 | 0 | 0 | 0 | 0 | 0 | 0 | 0 | 0 | 0 | 0 | 0 |
| Lb18 | 3 | 0 | 0 | 0 | 0 | 0 | 0 | 0 | 1 | 0 | 1 | 0 | 0 | 0 | 0 | 1 | 0 | 0 | 0 | 0 | 0 | 0 | 0 | 0 | 0 | 0 | 0 | 0 | 0 | 0 | 0 | 0 | 0 | 0 | 0 | 0 | 0 | 0 | 0 | 0 | 0 | 0 | 0 | 0 | 0 | 0 | 0 | 0 | 0 |
| Lb19 | 1 | 0 | 0 | 0 | 0 | 0 | 0 | 0 | 0 | 0 | 1 | 0 | 0 | 0 | 0 | 0 | 0 | 0 | 0 | 0 | 0 | 0 | 0 | 0 | 0 | 0 | 0 | 0 | 0 | 0 | 0 | 0 | 0 | 0 | 0 | 0 | 0 | 0 | 0 | 0 | 0 | 0 | 0 | 0 | 0 | 0 | 0 | 0 | 0 |
| Lb20 | 28 | 0 | 0 | 0 | 0 | 0 | 0 | 4 | 1 | 0 | 0 | 0 | 0 | 0 | 1 | 1 | 1 | 0 | 0 | 0 | 0 | 0 | 0 | 0 | 0 | 0 | 0 | 0 | 0 | 0 | 1 | 3 | 0 | 4 | 1 | 0 | 1 | 0 | 0 | 0 | 0 | 0 | 1 | 0 | 1 | 4 | 2 | 0 | 2 |
| Lb21 | 1 | 0 | 0 | 0 | 0 | 0 | 0 | 1 | 0 | 0 | 0 | 0 | 0 | 0 | 0 | 0 | 0 | 0 | 0 | 0 | 0 | 0 | 0 | 0 | 0 | 0 | 0 | 0 | 0 | 0 | 0 | 0 | 0 | 0 | 0 | 0 | 0 | 0 | 0 | 0 | 0 | 0 | 0 | 0 | 0 | 0 | 0 | 0 | 0 |
| Lb22 | 3 | 0 | 0 | 0 | 0 | 0 | 0 | 0 | 0 | 0 | 0 | 0 | 0 | 0 | 0 | 0 | 0 | 0 | 0 | 0 | 0 | 0 | 0 | 0 | 0 | 0 | 0 | 1 | 0 | 0 | 0 | 0 | 0 | 0 | 0 | 0 | 0 | 0 | 0 | 0 | 0 | 0 | 0 | 0 | 0 | 0 | 2 | 0 | 0 |
| Lb23 | 2 | 0 | 0 | 0 | 0 | 0 | 0 | 0 | 0 | 0 | 0 | 1 | 1 | 0 | 0 | 0 | 0 | 0 | 0 | 0 | 0 | 0 | 0 | 0 | 0 | 0 | 0 | 0 | 0 | 0 | 0 | 0 | 0 | 0 | 0 | 0 | 0 | 0 | 0 | 0 | 0 | 0 | 0 | 0 | 0 | 0 | 0 | 0 | 0 |
| Lb24 | 1 | 0 | 0 | 0 | 0 | 0 | 0 | 0 | 0 | 0 | 0 | 0 | 0 | 0 | 0 | 0 | 0 | 0 | 0 | 0 | 0 | 0 | 0 | 1 | 0 | 0 | 0 | 0 | 0 | 0 | 0 | 0 | 0 | 0 | 0 | 0 | 0 | 0 | 0 | 0 | 0 | 0 | 0 | 0 | 0 | 0 | 0 | 0 | 0 |
| Lb25 | 1 | 0 | 0 | 0 | 0 | 0 | 0 | 0 | 0 | 0 | 0 | 0 | 1 | 0 | 0 | 0 | 0 | 0 | 0 | 0 | 0 | 0 | 0 | 0 | 0 | 0 | 0 | 0 | 0 | 0 | 0 | 0 | 0 | 0 | 0 | 0 | 0 | 0 | 0 | 0 | 0 | 0 | 0 | 0 | 0 | 0 | 0 | 0 | 0 |
| Lb26 | 23 | 0 | 0 | 0 | 0 | 0 | 0 | 0 | 0 | 0 | 3 | 0 | 1 | 2 | 0 | 0 | 0 | 0 | 1 | 0 | 1 | 0 | 0 | 0 | 0 | 1 | 0 | 0 | 0 | 0 | 1 | 1 | 0 | 0 | 1 | 1 | 1 | 1 | 0 | 0 | 0 | 2 | 2 | 1 | 2 | 0 | 1 | 0 | 0 |
| Lb27 | 1 | 0 | 0 | 0 | 0 | 0 | 0 | 0 | 0 | 0 | 0 | 0 | 1 | 0 | 0 | 0 | 0 | 0 | 0 | 0 | 0 | 0 | 0 | 0 | 0 | 0 | 0 | 0 | 0 | 0 | 0 | 0 | 0 | 0 | 0 | 0 | 0 | 0 | 0 | 0 | 0 | 0 | 0 | 0 | 0 | 0 | 0 | 0 | 0 |
| Lb28 | 7 | 0 | 0 | 0 | 0 | 0 | 0 | 0 | 0 | 0 | 0 | 3 | 0 | 0 | 0 | 0 | 0 | 0 | 0 | 0 | 0 | 0 | 0 | 0 | 0 | 0 | 0 | 0 | 0 | 0 | 0 | 0 | 0 | 0 | 1 | 1 | 1 | 0 | 1 | 0 | 0 | 0 | 0 | 0 | 0 | 0 | 0 | 0 | 0 |
| Lb29 | 1 | 0 | 0 | 0 | 0 | 0 | 0 | 0 | 0 | 0 | 0 | 0 | 0 | 0 | 0 | 0 | 0 | 0 | 0 | 0 | 0 | 0 | 0 | 0 | 0 | 0 | 0 | 0 | 0 | 0 | 0 | 0 | 0 | 0 | 0 | 0 | 0 | 0 | 0 | 1 | 0 | 0 | 0 | 0 | 0 | 0 | 0 | 0 | 0 |
| Lb30 | 2 | 0 | 0 | 0 | 0 | 0 | 0 | 0 | 0 | 0 | 0 | 0 | 0 | 0 | 0 | 0 | 0 | 0 | 0 | 0 | 0 | 0 | 0 | 0 | 0 | 0 | 0 | 0 | 0 | 0 | 0 | 0 | 0 | 0 | 0 | 0 | 0 | 0 | 0 | 0 | 0 | 0 | 0 | 0 | 1 | 1 | 0 | 0 | 0 |
| Lb31 | 1 | 0 | 0 | 0 | 0 | 0 | 0 | 0 | 0 | 0 | 0 | 0 | 0 | 0 | 0 | 0 | 0 | 0 | 0 | 0 | 0 | 0 | 0 | 0 | 0 | 0 | 0 | 0 | 0 | 0 | 0 | 0 | 0 | 0 | 1 | 0 | 0 | 0 | 0 | 0 | 0 | 0 | 0 | 0 | 0 | 0 | 0 | 0 | 0 |
| Lb32 | 1 | 0 | 0 | 0 | 0 | 0 | 0 | 0 | 0 | 0 | 0 | 0 | 0 | 0 | 0 | 0 | 0 | 0 | 0 | 0 | 0 | 0 | 0 | 0 | 0 | 0 | 0 | 0 | 0 | 0 | 0 | 0 | 0 | 0 | 0 | 0 | 0 | 0 | 0 | 0 | 0 | 1 | 0 | 0 | 0 | 0 | 0 | 0 | 0 |
| Lb33 | 3 | 0 | 0 | 0 | 0 | 0 | 0 | 3 | 0 | 0 | 0 | 0 | 0 | 0 | 0 | 0 | 0 | 0 | 0 | 0 | 0 | 0 | 0 | 0 | 0 | 0 | 0 | 0 | 0 | 0 | 0 | 0 | 0 | 0 | 0 | 0 | 0 | 0 | 0 | 0 | 0 | 0 | 0 | 0 | 0 | 0 | 0 | 0 | 0 |
| Lb34 | 5 | 0 | 0 | 0 | 0 | 0 | 0 | 0 | 0 | 0 | 0 | 1 | 0 | 0 | 0 | 0 | 0 | 1 | 1 | 1 | 0 | 0 | 0 | 0 | 0 | 0 | 0 | 0 | 1 | 0 | 0 | 0 | 0 | 0 | 0 | 0 | 0 | 0 | 0 | 0 | 0 | 0 | 0 | 0 | 0 | 0 | 0 | 0 | 0 |
| Lb35 | 1 | 0 | 0 | 0 | 0 | 0 | 0 | 0 | 0 | 0 | 0 | 0 | 0 | 0 | 0 | 0 | 0 | 0 | 0 | 0 | 0 | 0 | 0 | 0 | 0 | 0 | 1 | 0 | 0 | 0 | 0 | 0 | 0 | 0 | 0 | 0 | 0 | 0 | 0 | 0 | 0 | 0 | 0 | 0 | 0 | 0 | 0 | 0 | 0 |
| Lb36 | 4 | 0 | 0 | 0 | 0 | 0 | 0 | 0 | 0 | 0 | 0 | 0 | 0 | 0 | 0 | 0 | 0 | 0 | 0 | 0 | 1 | 0 | 0 | 0 | 0 | 0 | 0 | 0 | 1 | 0 | 0 | 0 | 0 | 0 | 0 | 0 | 0 | 0 | 1 | 0 | 0 | 0 | 0 | 0 | 0 | 0 | 0 | 1 | 0 |
| Lb37 | 1 | 0 | 0 | 0 | 0 | 0 | 0 | 0 | 1 | 0 | 0 | 0 | 0 | 0 | 0 | 0 | 0 | 0 | 0 | 0 | 0 | 0 | 0 | 0 | 0 | 0 | 0 | 0 | 0 | 0 | 0 | 0 | 0 | 0 | 0 | 0 | 0 | 0 | 0 | 0 | 0 | 0 | 0 | 0 | 0 | 0 | 0 | 0 | 0 |

Supplement Table 4: Distribution of haplotypes of *L*. *tatei* for all localities. The sum of haplotypes for each locality equals the number of specimens studied form each locality. The haplotype names correspond to Fig. 2 and the locality names to Fig.1 and Table 1.

|  | locality | 2 | 15 | 20 | 23 | 25 | 28 | 31 | 32 | 34 | 35 | 36 | 43 | 44 | 51 | 52 | 65 |
| --- | --- | --- | --- | --- | --- | --- | --- | --- | --- | --- | --- | --- | --- | --- | --- | --- | --- |
| Haplotype number | Sum of haplotypes | 1 | 10 | 5 | 4 | 6 | 4 | 2 | 5 | 1 | 3 | 2 | 1 | 1 | 1 | 1 | 1 |
| Lt1 | 1 | 0 | 0 | 0 | 0 | 1 | 0 | 0 | 0 | 0 | 0 | 0 | 0 | 0 | 0 | 0 | 0 |
| Lt2 | 33 | 0 | 8 | 4 | 3 | 1 | 3 | 1 | 4 | 1 | 3 | 1 | 1 | 1 | 1 | 1 | 0 |
| Lt3 | 1 | 0 | 0 | 0 | 0 | 0 | 0 | 1 | 0 | 0 | 0 | 0 | 0 | 0 | 0 | 0 | 0 |
| Lt4 | 7 | 0 | 0 | 1 | 0 | 3 | 0 | 0 | 1 | 0 | 0 | 1 | 0 | 0 | 0 | 0 | 1 |
| Lt5 | 1 | 0 | 1 | 0 | 0 | 0 | 0 | 0 | 0 | 0 | 0 | 0 | 0 | 0 | 0 | 0 | 0 |
| Lt6 | 3 | 0 | 1 | 0 | 1 | 1 | 0 | 0 | 0 | 0 | 0 | 0 | 0 | 0 | 0 | 0 | 0 |
| Lt7 | 1 | 0 | 0 | 0 | 0 | 0 | 1 | 0 | 0 | 0 | 0 | 0 | 0 | 0 | 0 | 0 | 0 |
| Lt8 | 1 | 1 | 0 | 0 | 0 | 0 | 0 | 0 | 0 | 0 | 0 | 0 | 0 | 0 | 0 | 0 | 0 |

Supplement Table 5: Distribution of haplotypes of *L*. *paratatei* for all localities. The sum of haplotypes for each locality equals the number of specimens studied form each locality. The haplotype names correspond to Fig. 2 and the locality names to Fig.1 and Table 1.

|  | locality | 17 | 18 | 39 | 41 | 42 | 43 | 54 |
| --- | --- | --- | --- | --- | --- | --- | --- | --- |
| Haplotype number | Sum of haplotypes | 3 | 5 | 9 | 12 | 5 | 4 | 5 |
| Lc1 | 14 | 1 | 3 | 2 | 7 | 0 | 0 | 1 |
| Lc2 | 1 | 1 | 0 | 0 | 0 | 0 | 0 | 0 |
| Lc3 | 3 | 0 | 1 | 0 | 0 | 0 | 0 | 2 |
| Lc4 | 16 | 1 | 1 | 2 | 5 | 5 | 2 | 0 |
| Lc5 | 4 | 0 | 0 | 1 | 0 | 0 | 1 | 2 |
| Lc6 | 5 | 0 | 0 | 4 | 0 | 0 | 1 | 0 |
